# The BRCA1/BARD1 complex recognizes pre-ribosomal RNA to facilitate homologous recombination

**DOI:** 10.1038/s41421-023-00590-8

**Published:** 2023-10-03

**Authors:** Duo Wu, Huang Huang, Tenglong Chen, Xiaochen Gai, Qilin Li, Chunhui Wang, Jia Yao, Yu Liu, Shang Cai, Xiaochun Yu

**Affiliations:** 1 Westlake Laboratory of Life Sciences and Biomedicine, Hangzhou, Zhejiang China; 2 School of Life Sciences, Westlake University, Hangzhou, Zhejiang China; 3 Institute of Basic Medical Sciences, Westlake Institute for Advanced Study, Hangzhou, Zhejiang China; 4 Westlake Disease Modeling Lab, Westlake Laboratory of Life Sciences and Biomedicine, Hangzhou, Zhejiang China; 5 Key Laboratory of Growth Regulation and Translational Research of Zhejiang Province, School of Life Sciences, Westlake University, Hangzhou, Zhejiang China; 6 The First Affiliated Hospital, School of Medicine, Zhejiang University, Hangzhou, Zhejiang China

**Keywords:** Homologous recombination, Mechanisms of disease

## Abstract

The BRCA1/BARD1 complex plays a key role in the repair of DNA double-strand breaks (DSBs) in both somatic cells and germ cells. However, the underlying molecular mechanism by which this complex mediates DSB repair is not fully understood. Here, we examined the XY body of male germ cells, where DSBs are accumulated. We show that the recruitment of the BRCA1/BARD1 complex to the unsynapsed axis of the XY body is mediated by pre-ribosomal RNA (pre-rRNA). Similarly, the BRCA1/BARD1 complex associates with pre-rRNA in somatic cells, which not only forms nuclear foci in response to DSBs, but also targets the BRCA1/BARD1 complex to DSBs. The interactions between the BRCT domains of the BRCA1/BARD1 complex and pre-rRNA induce liquid–liquid phase separations, which may be the molecular basis of DSB-induced nuclear foci formation of the BRCA1/BARD1 complex. Moreover, cancer-associated mutations in the BRCT domains of *BRCA1* and *BARD1* abolish their interactions with pre-rRNA. Pre-rRNA also mediates BRCA1-dependent homologous recombination, and suppression of pre-rRNA biogenesis sensitizes cells to PARP inhibitor treatment. Collectively, this study reveals that pre-rRNA is a functional partner of the BRCA1/BARD1 complex in the DSB repair.

## Introduction

Breast cancer susceptibility gene 1 (*BRCA1*) is an important tumor suppressor gene, as mutations of *BRCA1* are associated with 20%–30% of familial breast and ovarian cancers^1,2^. The gene product plays a key role in DNA double-strand break (DSB) repair. Mutations of *BRCA1* abolish its function in the DSB repair, causing genomic instability and tumorigenesis^3,4^.

BRCA1 is an 1863-residue nuclear polypeptide with a prominent N-terminal Ring domain and an evolutionarily conserved C-terminal BRCT domain^5^. The Ring domain of BRCA1 interacts with the Ring domain of BRCA1 Associated Ring Domain 1 (BARD1), which forms a BRCA1/BARD1 heterodimer^6^. The C-terminal BRCT domain consists of two tandem BRCT repeats and acts as a phospho-Ser binding domain^7–9^. One of the major functions of the BRCT domain is to target BRCA1 to DSBs for lesion repair. Consistently, this BRCT domain alone can relocate to DSBs^10^. Since BRCA1 participates in several key steps of homologous recombination (HR)^4,11^, one major mechanism for DSBs repair, loss of the BRCT domain impairs HR repair. Numerous missense mutations in the BRCT domain have been identified in familial breast and ovarian cancer patients, indicating that the BRCT domain plays a key role to maintain genomic stability. Most of these cancer-associated missense mutations abolish the phosphate moiety binding capability, resulting in defective recruitment of BRCA1 to DNA lesions for HR repair^12,13^. We and others have identified several binding partners of this BRCT domain, including ABRAXAS1^14–16^, BRIP1^7,17^, and CTIP^18,19^. Interactions between BRCA1 and these binding partners form BRCA1 A, B, C complexes, respectively^20^. Although these BRCT domain-binding partners participate in the DSB repair, none of these known partners can mediate the relocation of BRCA1 to DSBs, suggesting that other yet unidentified partner(s) of the BRCA1 BRCT domain may recruit BRCA1 to DSBs for HR repair.

Similar to the domain architecture of BRCA1, BARD1 also contains a C-terminal BRCT domain. And like the BRCA1 BRCT domain, the BARD1 BRCT domain has a putative phosphate moiety-binding pocket^10,21^. However, phospho-protein-binding partner(s) of the BARD1 BRCT domain has yet to be identified. Interestingly, the BARD1 BRCT domain recognizes poly(ADP-ribosyl)ation, a posttranslational modification induced by DNA damage^10^. Since poly-ADP ribose is a special type of nucleic acid chain and contains numerous phosphate moieties, the BARD1 BRCT domain may recognize the phosphate groups in nucleic acid^22^. Unlike the Ring domain, the BRCT domain of BARD1 does not interact with BRCA1. But similar to the BRCA1 BRCT domain, it also recognizes DSBs^10^. Moreover, like the BRCA1 BRCT domain, cancer-associated missense mutations have also been identified in the BARD1 BRCT domain^23,24^, suggesting that this BARD1 BRCT domain may also play an important role in maintaining genomic stability and suppression of tumorigenesis. The BRCA1 and BARD1 form a heterodimer in nucleus^6,25^. Disruption of either one in this complex induces degradation of the other subunit^26^, further indicating that BRCA1 and BARD1 act together in HR repair.

Similar to DSB repair in somatic cells, the BRCA1/BARD1 complex also participates in HR in germ cells, in which SPO11 induces DSBs for genomic DNA exchange between maternal and paternal chromosomes^3,27,28^. Interestingly, in male germ cells, the sex chromosomes have little homology and thus cannot pair together except pseudo homologous regions close to telomere^28^. However, the X and Y chromosomes can still be cut by SPO11, and numerous DNA damage repair (DDR) factors are loaded onto these two sex chromosomes during meiotic prophase^28^. Thus, DDR factors and these two chromosomes form a unique liquid–liquid phase separation (LLPS) named the XY body in male germ cells during meiotic prophase, which perhaps is the largest DSB-induced focus in mammalian cells^28^. Inside of this nuclear body, BRCA1 localizes at the unsynapsed region of the X and Y chromosomes, and colocalizes with RAD51, a key enzyme for HR repair, indicating that similar to somatic cells, BRCA1 may also mediate DSB repair in germ cells^28–31^. However, how BRCA1 is recruited to the unsynapsed region of the X and Y chromosomes is unclear.

Interestingly, in addition to the known DSB repair factors, our recent study showed that pre-ribosomal RNA (pre-rRNA) also localizes at the unsynapsed region^32^. In-depth analyses showed that MDC1, an upstream mediator binding to γH2AX at DSBs, recognizes pre-rRNA and loads pre-rRNA to DSBs, suggesting that pre-rRNA directly participates in DSB repair^32^. Since RNA species often act as scaffolds to maintain LLPS in subnuclear bodies, it is possible that pre-rRNA maintains DNA damage-induced foci in both male germ cells and somatic cells^32^. However, the functional interactions between the BRCA1/BARD complex and pre-rRNA at DNA lesions remain elusive. Here, we examined both male germ cells and somatic cells, and found that pre-rRNA mediates the recruitment of the BRCA1/BARD1 complex to DSBs for HR repair.

## Results

### The BRCA1/BARD1 complex recognizes RNA species in the XY body and ionizing radiation-induced foci (IRIF)

**Fig. 1:**
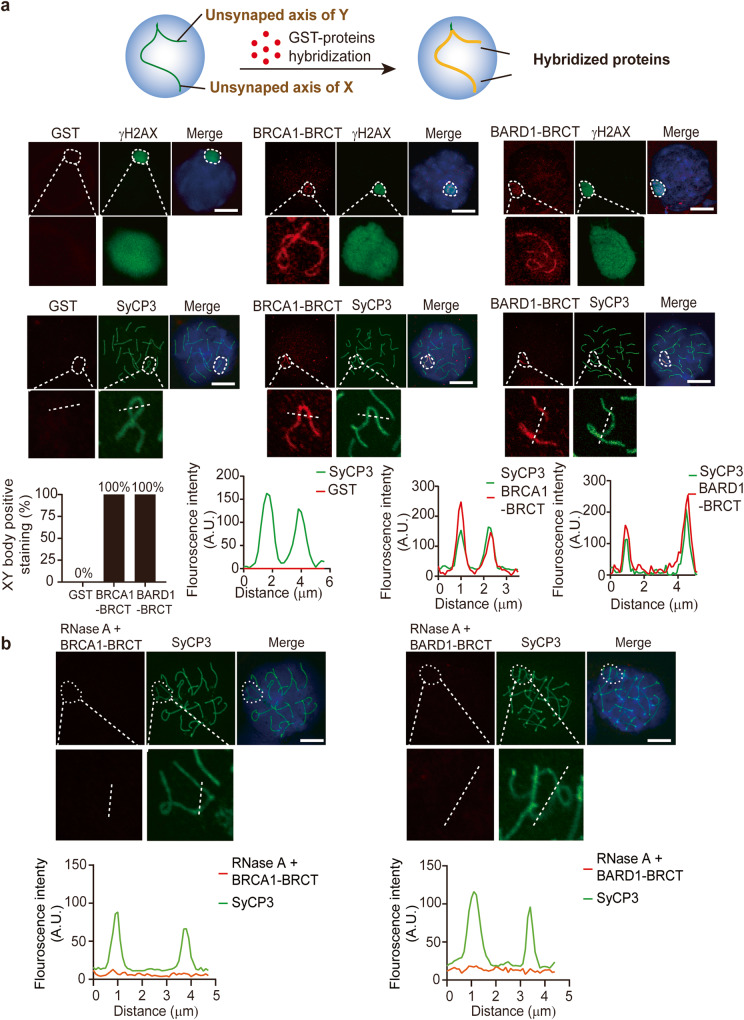

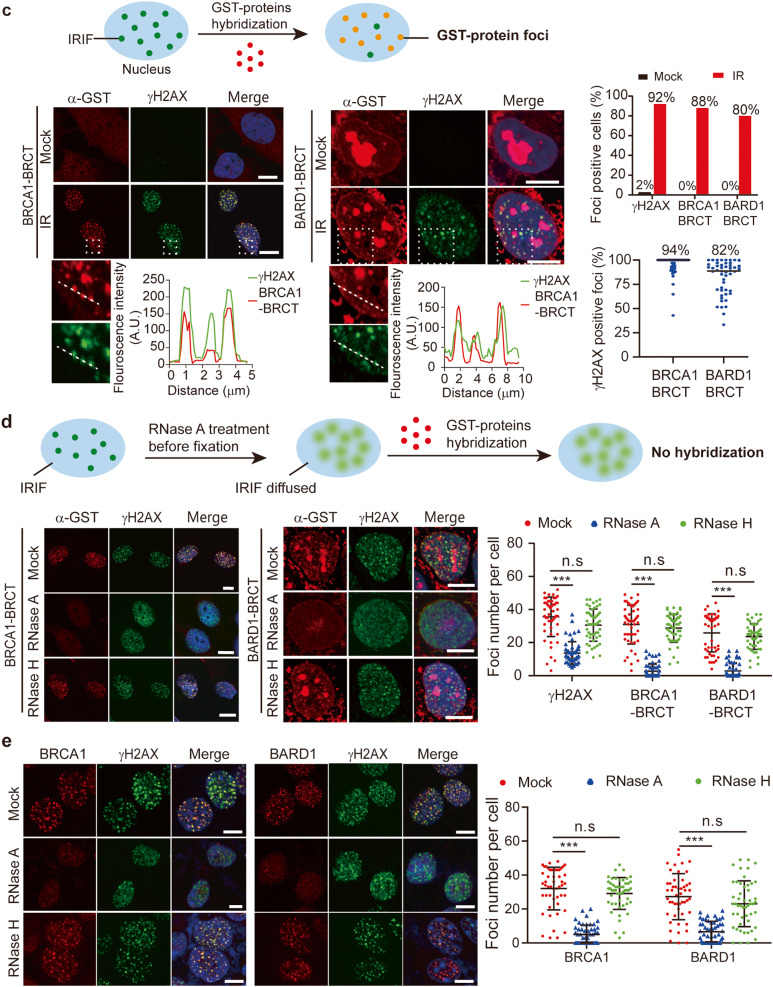
The BRCA1/BARD1 complex recognizes RNA species in the XY body and IRIF. **a** Incubation of recombinant BRCA1 BRCT or BARD1 BRCT with meiotic spreads. γH2AX is a surrogate marker of the XY body. SyCP3 is a surrogate marker of the unsynapsed axis of X and Y chromosomes. Colocalization of the BRCA1 BRCT and BARD1 BRCT with SyCP3 on the unsynapsed axis was examined. Fluorescence intensity is analyzed on the dashed line. **b** Meiotic spreads were pre-treated with RNase A prior to the incubation with the recombinant BRCA1 BRCT or BARD1 BRCT. Colocalization of the BRCA1 BRCT and BARD1 BRCT with SyCP3 on the unsynapsed axis was examined. Fluorescence intensity is analyzed. **c** Incubation IR-treated U2OS cells with recombinant BRCA1 BRCT or BARD1 BRCT. U2OS cells were treated with or without 10 Gy of IR. After a 12-h recovery, the cells were incubated with the recombinant proteins. Recombinant proteins were labeled with anti-GST antibodies. DSBs were labeled with anti-γH2AX antibody. The fluorescence signals on dash lines were analyzed. The percentage of foci-positive cells (> 15 foci) was measured. **d** Following RNase A or RNase H treatment, the recombinant BRCA1 BRCT- or BARD1 BRCT- recognized IRIF was examined in U2OS cells. Foci number per cell was analyzed. **e** The IRIF of endogenous BRCA1 and BARD1 was examined in U2OS cells. Foci number per cell was analyzed. *P* values were calculated using Student’s *t*-test. n.s. nonsignificant, *P < 0.05, ***P < 0.001. Scale bars, 10 μm.

To examine the molecular mechanism of the BRCA1/BARD1 complex, we explored the localization of the BRCA1/BARD1 complex in the XY body of male germ cells. Earlier studies showed that BRCA1 colocalizes with RAD51 at the unsynapsed axis of the XY body^33^, suggesting that BRCA1 participates in DSB repair at the unsynapsed axis. Since the BRCT domains of BRCA1 and BARD1 target the complex to DSBs^7,10^, we generated recombinant GST-BRCA1 BRCT domain and GST-BARD1 BRCT domain (Supplementary Fig. S1), and incubated both BRCT domains with meiotic spreads. Using this protein-binding approach, we found that both BRCT domains of BRCA1 and BARD1 were able to recognize the unsynapsed axis, suggesting that the BRCT domain recognizes a functional partner at the unsynapsed region (Fig. 1a). Since the BRCT domain is known as a phospho-Ser-binding motif, we ask if a phospho-protein mediates the recruitment of BRCA1 to the unsynapsed region. We treated meiotic spreads with l phosphatase to remove all protein phosphorylation marks, such as γH2AX, a surrogate marker for the XY body and DSBs. However, this phosphatase treatment did not abolish the binding of the BRCT domains to the unsynapsed axis, suggesting that the BRCT domains recognize functional partner(s) other than phospho-proteins in this protein-binding assay (Supplementary Fig. S2).

Our earlier studies suggested that RNA species exist in the XY body, such as rRNA, snoRNA and RNase MRP^31^. Thus, we treated meiotic spreads with RNase A to remove RNA species. Interestingly, we found that the BRCT domains did not recognize the unsynapsed axis anymore (Fig. 1b), suggesting that the BRCT domains may recognize RNA species in the XY body. Similarly, since the BRCT domains mediate the recruitment of the BRCA1/BARD1 complex to DNA lesions, the BRCT domain alone is able to reach DSBs in somatic cells^10^. We treated U2OS cells with ionizing radiation (IR). When DSBs were induced following IR treatment and were marked by γH2AX, both recombinant BRCA1 BRCT and BARD1 BRCT localized onto the IRIF and colocalized with γH2AX or RAD51 (Fig. 1c and Supplementary Fig. S3), suggesting that these recombinant proteins can recognize IR-induced DSBs in this protein-binding setting. In contrast, ankyrin (ANK) repeats of BARD1 could not bind to IRIF (Supplementary Fig. S4). Again, RNase A treatment abolished the localization of the BRCT domains to DSBs, suggesting that the BRCT domains may recognize RNA species at the IR-induced DSBs in somatic cells (Fig. 1d). Moreover, the localization of this protein was not affected by RNase H treatment (Fig. 1d), excluding the possibility that R-loops mediate the recruitment of the BRCT domains to the IR-induced DSBs. Moreover, we examined IRIF of endogenous BRCA1 and BARD1. Again, with the RNase A but not RNase H treatment, the foci formation of BRCA1 and BARD1 was disrupted (Fig. 1e). Collectively, these results suggest that the BRCA1/BARD1 complex recognizes RNA species at SPO11-induced DSBs on the unsynapsed axis during meiotic prophase as well as at the IR-induced DSBs in somatic cells.

### The BRCT domains of BRCA1 and BARD1 bind to pre-rRNAs

To identify the BRCA1/BARD1 complex-associated RNA, we established 293T cells stably expressing SFB (an S-protein-binding tag, two tandem FLAG tags, and a Streptavidin-binding peptide tag)-tagged BARD1 or the BRCA1 BRCT domain alone. Ectopically expressed BARD1 or the BRCA1 BRCT domain was recruited to DSBs when cells were gamma-irradiated, suggesting that these ectopically expressed proteins are involved in DSB response (Supplementary Fig. S5a). Moreover, the expression levels of the exogenous BARD1 or the BRCA1 BRCT are similar to those of endogenous BARD1 or BRCA1 (Supplementary Fig. S5b, c).

To further validate these results, we expressed BRCA1 BRCT or BARD1 BRCT in MDA-MB-231 (BRCA1 proficient) or MDA-MB-436 (BRCA1 deficient) cells (Supplementary Fig. S6a). These BRCTs colocalized with RAD51 in response to IR (Supplementary Fig. S6b–d), suggesting that these BRCTs localize to DSBs. Moreover, we examined and found that the IR-induced foci of pre-rRNA largely colocalized with those of BRCTs (Supplementary Fig. S7). Finally, RNase A treatment largely abolished the foci of the BRCTs (Supplementary Fig. S8), suggesting that RNA mediates the recruitment of the BRCTs to DSBs.

**Fig. 2:**
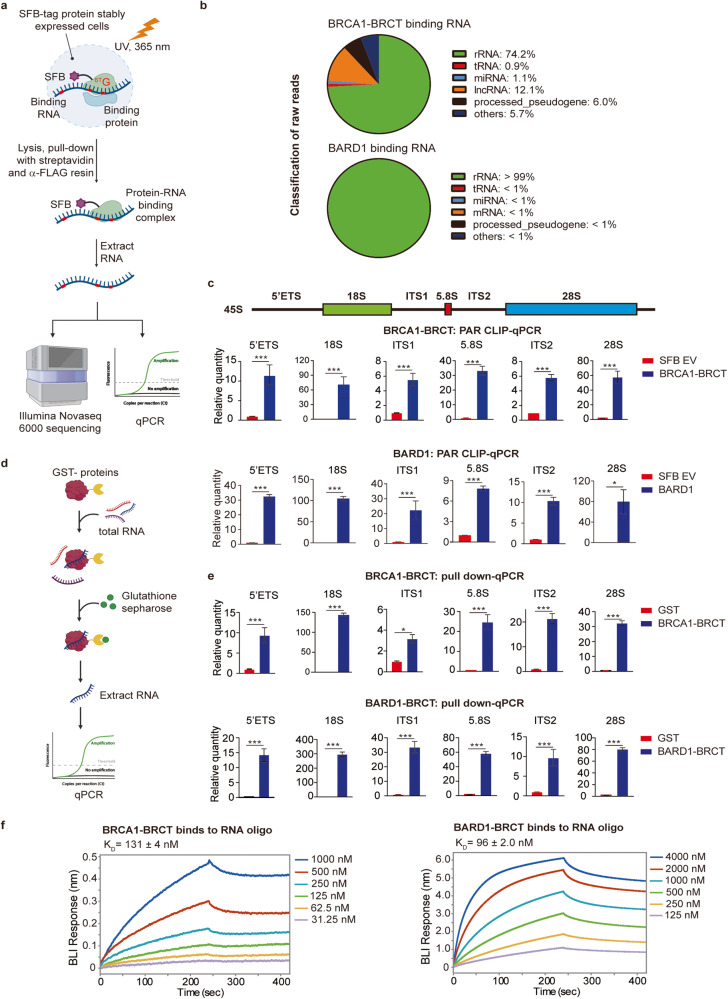
The BRCT domains of BRCA1 and BARD1 bind to pre-rRNAs. **a** Schematic diagram for identification of the BRCA1/BARD1 complex-associated RNA species. **b** Pie charts showing the BRCA1/BARD1 complex-associated RNA species. **c** Interactions between pre-rRNA and the BRCA1/BARD1 complex are validated by PAR-CLIP and RT-qPCR. **d**, **e** Interactions between the recombinant BRAC1-BRCT and BARD1 BRCT and pre-rRNA were examined by GST pull-down assays. GST, GST-BRCA1 BRCT, or GST-BARD1 BRCT proteins were incubated with pre-rRNA. Following GST pull-down assays, RT-qPCR was performed to examine the enrichment of pre-rRNA. Values are means ± SD of three independent assays. **f** The BRCT domains of BRCA1 and BARD1 directly interact with rRNA. Binding affinities between the BRCT domains and biotin-labeled RNA oligos (25nt) were measured by BLI assays.

Next, these cells were incubated with 6-thioguanosine (6-TG) for crosslinking RNA to its associated proteins. We then performed protein affinity purification and RNA sequencing (Fig. 2a). We used 293T cells only expressing the SFB tag as a negative control, and subtracted non-specific RNA in the control samples from the RNA found in the tested samples. Interestingly, the majority of RNA species associated with BARD1 or the BRCA1 BRCT domain were rRNA (Fig. 2b). We further validated these results using RT-qPCR with primers in every region of rDNA locus, where 45 S pre-rRNA is transcribed by RNA polymerase I (pol I). Interestingly, we found that RNA in the 18 S region was more selectively recognized by BARD1 or BRCA1 BRCT (Fig. 2c). Since the BRCT domains of BRCA1 and BARD1 may recognize RNA species at DNA lesions, we incubated GST-BRCA1 BRCT or GST-BARD1 BRCT with total RNA extracted from 293T cells, and performed RT-qPCR. Again, both BRCT domains recognized pre-rRNA, especially the 18 S region (Fig. 2d, e). To examine if there are direct bindings between the BRCT domains and pre-rRNA, we measured the binding affinity between the BRCT domains and RNA using biolayer interferometry (BLI) (Supplementary Fig. S9a). A highly enriched RNA sequence from the protein affinity purification and RNA sequencing results was chosen as RNA oligos (Supplementary Fig. S9b). Both BRCA1 BRCT and BARD1 BRCT domains have strong affinities with RNA oligos with dissociation constants at 131 nM and 96 nM, respectively (Fig. 2f). Moreover, we found that the BRCA1 BRCT domain recognized RNA with more than 10 nt, whereas the BARD1 BRCT domain bound to RNA as short as 3 nt (Supplementary Fig. S9c, d). Taken together, these results suggest that both BRCA1 BRCT domain and BARD1 BRCT domain recognize pre-rRNAs directly.

### The BRCA1/BARD1 complex is associated with ribosomal proteins

**Fig. 3:**
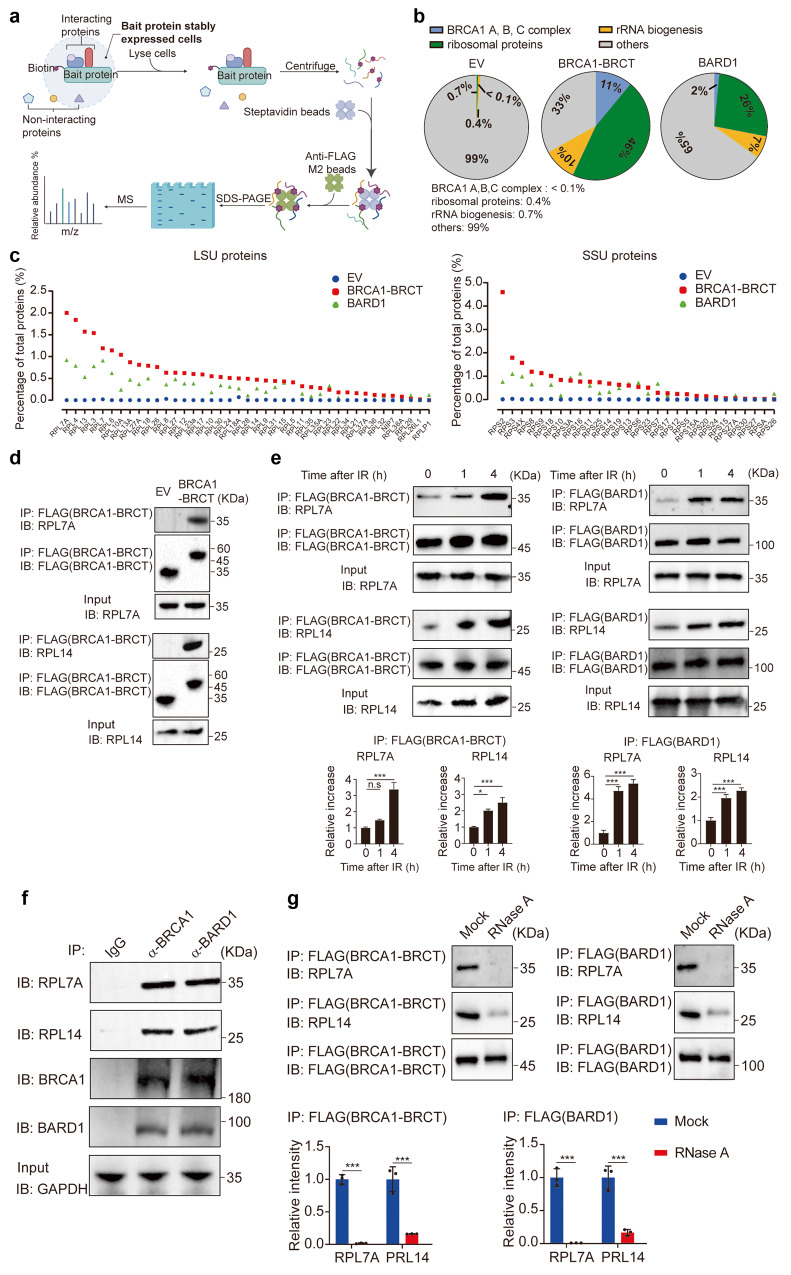
The BRCA1/BARD1 complex is associated with ribosomal proteins. **a** A diagram illustrating methods for a mass spectrometry proteomics experiment for the identification of the BRCA1- and BARD1-associated proteins. 293T stably expressing SFB–BRCA1 BRCT or SFB–BARD1 were used for the analyses. SFB empty vector (EV) was used as a negative control. **b** Pie charts depicting the binding partners of BRCA1 and BARD1 from mass spectrometry analysis. **c** A myriad of ribosomal proteins identified from the mass spectrometry analysis are shown. BRCA1 BRCT or BARD1-associated large subunit (LSU) and small subunit (SSU) proteins are shown separately. **d** The BRCA1 BRCT interacts with RPL7A and RPL14. 293T cells stably expressing BRCA1 BRCT were examined. Co-IP and western blotting assay were performed with indicated antibodies. **e** IR induces interactions between the BRCA1/BARD1 complex and ribosomal proteins. 293 T cells stably expressing BRCA1 BRCT or BARD1 were treated with 10 Gy of IR. Following the indicated recovering time, RPL7A and RPL14 were assessed by co-IP and western blotting assay. The BRCA1 BRCT- or BARD1-associated RPL7A or RPL14 are analyzed in the histograms (bottom panel). Values are means ± SD of three independent assays. *P* values were calculated using Student’s *t*-test. n.s. nonsignificant, *P < 0.05, ***P < 0.001. **f** Associations between the endogenous BRCA1/BARD1 complex and ribosomal proteins were examined by co-IP and western blotting assay with indicated antibodies. **g** The RNase A treatment abolishes the interactions between the BRCA1/BARD1 complex and ribosomal proteins. The cell lysates isolated from 293T cells stably expressing SFB–BRCA1 BRCT or SFB–BARD1 were treated with or without RNase A prior to IP. The associated RPL7A or RPL14 is summarized in the histograms (bottom panel). Values are means ± SD of three independent assays.

Using these stable cell lines, we performed tandem protein affinity purification and mass spectrometry analysis (Fig. 3a). Consistent with earlier reports, we found that BARD1 or the BRCA1 BRCT domain is associated with other protein subunits in the BRCA1 A, B, C complexes (Supplementary Table S1). However, in addition to the protein subunits of the BRCA1 A, B, C complexes, we found that BARD1 or the BRCA1 BRCT domain is associated with a large number of ribosomal proteins and proteins related to rRNA biogenesis (Fig. 3b). Although ribosomal proteins are abundantly expressed in the cell, little amount of these proteins was found in the control samples from cells transfected with empty vectors, indicating that the BRCA1/BARD1 complex may selectively associate with ribosomal protein subunits.

To date, there are 80 known ribosomal proteins in human cells. We further examined and found that a large portion of these ribosomal proteins are associated with the BRCA1 BRCT domain or BARD1 (Fig. 3c). Next, we randomly selected RPL7A and RPL14 and validated the interactions using co-immunoprecipitation (co-IP) assays (Fig. 3d). Interestingly, the interactions between the BRCA1 BRCT domain and ribosomal proteins were clearly increased following IR treatment to induce DSBs. Similarly, DSBs also promoted the association between BARD1 and ribosomal proteins (Fig. 3e). Moreover, the interactions between the endogenous BRCA1/BARD1 complex and the ribosomal proteins were confirmed with co-IP assays (Fig. 3f). Collectively, these results demonstrate that the BRCA1/BARD1 complex is associated with ribosomal proteins, which are further enhanced in response to DSBs.

We ask if the BRCT domains of BRCA1 and BARD1 recognize phosphorylated ribosomal proteins. The IPed materials were treated with l phosphatase to digest protein phosphorylation. However, this phosphatase treatment did not disrupt the interactions between ribosomal proteins and the BRCA1 BRCT domain or BARD1 (Supplementary Fig. S10). Next, the IPed substances were incubated with RNase A to remove RNA species. The interactions between the ribosomal proteins and the BRCT domain or BARD1 were disrupted following the RNase A treatment (Fig. 3g), suggesting that the interactions between the BRCA1/BARD1 complex and ribosomal proteins are mediated by pre-rRNA.

### Pre-rRNA targets the BRCA1/BARD1 complex to DSBs

**Fig. 4:**
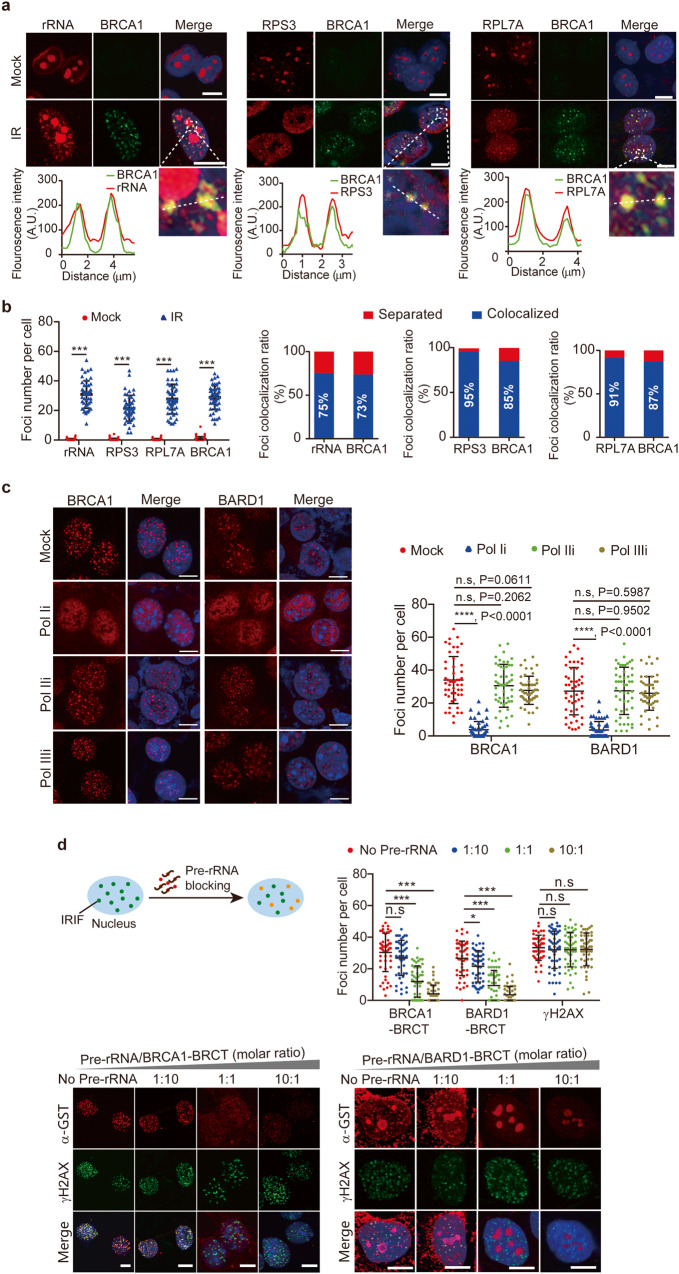

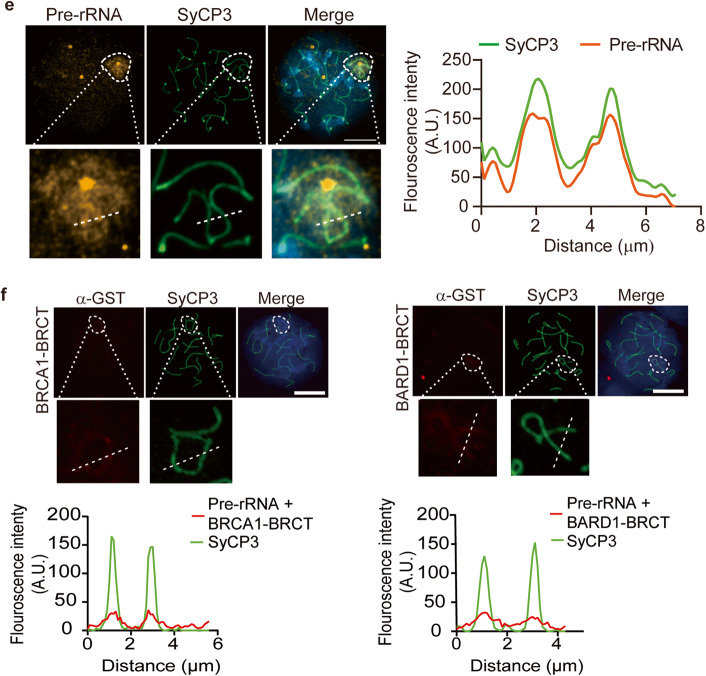
Pre-rRNA targets the BRCA1/BARD1 complex to DSBs. **a** Pre-rRNP exists at IRIF. Cells were treated with 10 Gy of IR. Pre-rRNA was examined by RNA probes. Ribosomal proteins were stained with indicated antibodies. The colocalization with BRCA1 was analyzed (bottom panel). **b** Foci number per cell and foci colocalization ratio are examined. **c** The IRIF of the BRCA1/BARD1 complex in the pol Ii-treated cells. HeLa cells were treated with RNA polymerase inhibitors prior to 10 Gy of IR. The IRIF of BRCA1 and BARD1 were stained with indicated antibodies. Foci number per cell is shown (right panel). The cells were also counterstained by DAPI. *P* values were calculated using Student’s *t*-test. Scale bars, 10 μm. **d** A schematic diagram showing that pre-incubation with pre-rRNA blocks the binding of the recombinant BRCA1 BRCT or BARD1 BRCT to IRIF (top left panel). Foci were examined with anti-GST antibody (bottom panel). DSB foci were marked with an anti-γH2AX antibody. Foci number per cell is shown (top right panel). *P* values were calculated using Student’s *t* test. n.s. nonsignificant, *P < 0.05, ***P < 0.001. Scale bars, 10 μm. **e** Enrichment of pre-rRNA at the unsynapsed axis of X and Y chromosomes. Analysis of pre-rRNA and SCP3 distribution is shown (right panel). **f** Pre-incubation of the BRCTs with pre-rRNA abolishes their localization onto the XY body. Recombinant BRCT proteins were pre-incubated with pre-rRNA. The circled area indicates the XY body. Scale bars, 10 μm.

To explore the possible role of pre-rRNA in DSB repair, we examined the localization of pre-rRNA. In the mock-treated cells, pre-rRNA only existed in nucleoli. However, following IR treatment to induce DSBs, pre-rRNA was recruited to DSBs and colocalized with BRCA1 (Fig. 4a, b). In addition, pre-rRNA-associated proteins, such as ribosomal protein subunits RPL7A and RPS3, also contributed to the formation of IR-induced foci (Fig. 4a, b).

Since the BRCA1/BARD1 complex associated with pre-rRNA, we examined IRIF of this pre-ribosomal ribonucleoprotein (pre-rRNP) complex in MDA-MB-231 (BRCA1 proficient) and MDA-MB-436 (BRCA1 deficient) cells, and found that pre-rRNP foci formed in both cells (Supplementary Fig. S11), indicating that BRCA1 is not required for the relocation of the pre-rRNP complex to DSBs. Next, we treated cells with RNA pol-I inhibitor to transiently suppress the biogenesis of pre-rRNA. Interestingly, the pol-I inhibitor treatment suppressed the IRIF of BRCA1 and BARD1 (Fig. 4c). Moreover, since the BRCT domains target the BRCA1/BARD1 complex to DNA lesions, we ask if the BRCT domains recognize pre-rRNA at DSBs. We have shown that both BRCA1 BRCT and BARD1 BRCT can relocate to DSBs (Fig. 1c). When we extracted pre-rRNA and incubated pre-rRNA with recombinant BRCT domains, both recombinant proteins failed to recognize DSBs in the presence of excessive pre-rRNA (Fig. 4d). In addition to the IRIF, pre-rRNA localized at the unsynapsed region of the XY body, where SPO11-induced DSBs occur (Fig. 4e). Pre-incubation of pre-rRNA with BRCT domains also blocked the localization of the BRCT domains to the unsynapsed region (Fig. 4f). All together, these results suggest that pre-rRNA may mediate the recruitment of the BRCA1/BARD1 complex to DSBs.

### The BRCT domains and pre-rRNA mediate phase separation

**Fig. 5:**
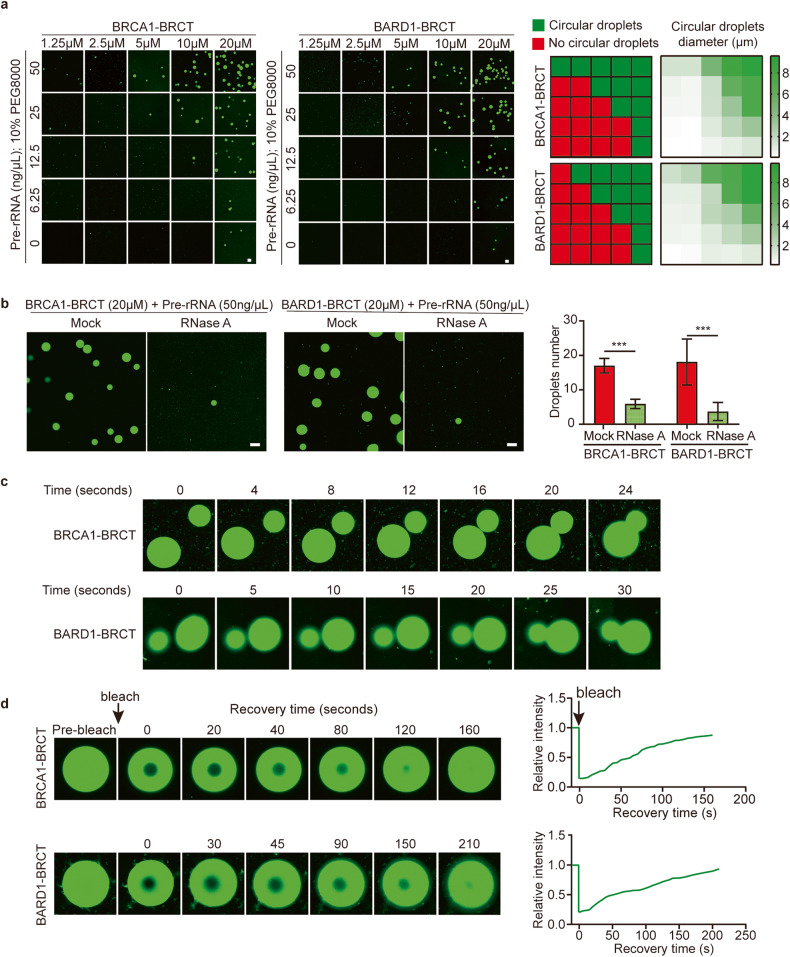
The BRCT domains and pre-rRNA form LLPS. **a** The BRCT domains of BRCA1 or BARD1 form LLPS with pre-rRNA. Recombinant BRCA1 BRCT or BARD1 BRCT was incubated with pre-rRNA in the presence of a crowding agent (10% PEG 8000). LLPS was examined by microscopy after incubation on a coverslip for 10 min. Droplet formation and average droplet diameter were analyzed (right panels). Corresponding square networks consist of green and red dots representing droplet formation in indicated conditions. **b** LLPS of the BRCTs and pre-rRNA complex was abolished by RNase A treatment. Recombinant BRCA1 BRCT and BARD1 BRCT were incubated with pre-rRNA, followed by 1 mg/mL RNase A treatment. Statistical analysis of droplet formation is shown (right panel). Values are means ± SD of three independent assays. **c** Microscopy images of individual droplet fusions at indicated time points. **d** Microscopy images of individual droplet dynamic imaging at indicated time points followed by photobleaching. Statistical analysis of droplets fluorescence is shown (right panel). Scale bars, 10 μm.

Both IRIF and the XY body are unique types of LLPS in nucleus. Since the BRCT domains recognize pre-rRNA and target the BRCA1/BARD complex to DSBs, we ask if the BRCT domains and pre-rRNA form LLPS. We incubated recombinant GFP-tagged BRCT domains with pre-rRNA. With the increased concentrations, BRCTs and pre-rRNA started to form phase separation droplets in vitro (Fig. 5a). Next, when we treated the droplets with RNase A to digest pre-rRNA, the droplets were disassembled (Fig. 5b), indicating that pre-rRNA is a key component of LLPS droplets formed with the BRCT domains. Moreover, recording the droplet fusion process and fluorescence bleaching and recovering process indicated that droplet formation is a dynamically growing process (Fig. 5c, d). Collectively, these results suggest that the BRCT domains of the BRCA1/BARD1 complex and pre-rRNA form LLPS in vitro, which could be the molecular basis of IRIF of BRCA1 and BARD1 in cells.

### Cancer-associated mutations in the BRCT domains abolish their interactions with pre-rRNA, LLPS, and IRIF

**Fig. 6:**
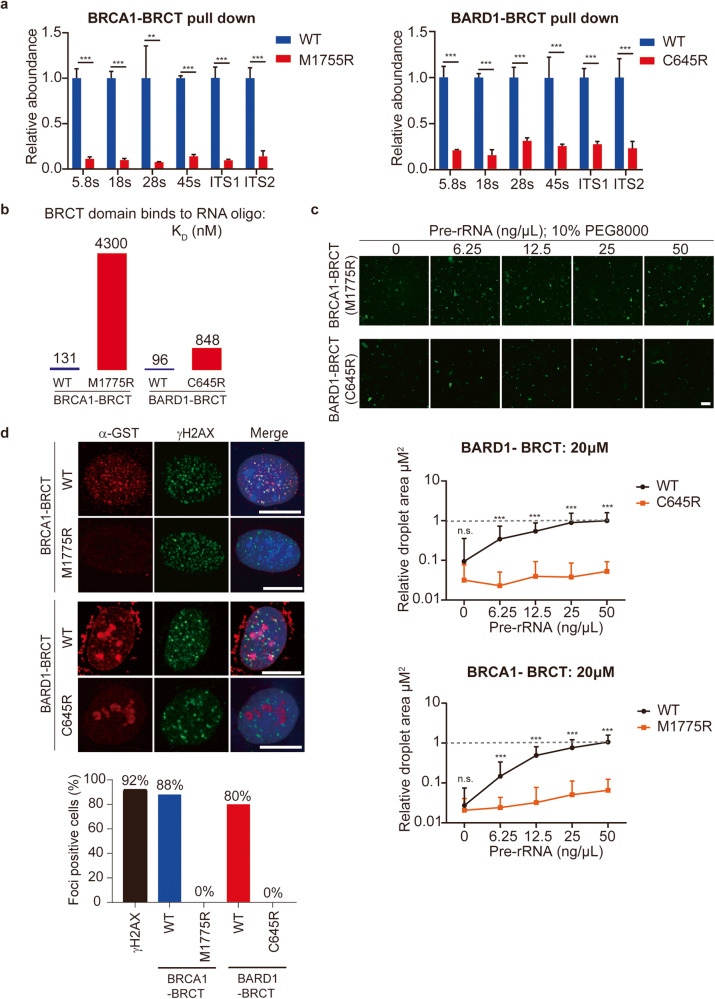
Cancer-associated mutations in the BRCT domains abolish the interactions with pre-rRNA. **a** The association between pre-rRNA and M1755R of the BRCA1 BRCT or C645R of the BARD1 BRCT was examined by protein pull-down assays. RT-qPCR was performed following the GST-protein pull-down assays. **b** The mutations of the BRCT domains drastically reduce their binding affinity with RNA. The binding affinities between the mutant BRCT domains and 25-nt biotin-labeled RNA oligos were measured by BLI assays. **c** The BRCT domain mutants fail to form LLPS droplets even in the presence of pre-rRNA. The droplet area was measured. Wild-type BRCTs forming droplets in the presence of 50 ng/µL pre-RNA was used as control. **d** The BRCT domain mutants fail to localize onto IRIF. Foci-positive cells were analyzed. Scale bars, 10 μm.

Cancer-associated missense mutations are often found in BRCT domains of *BRCA1* and *BARD1*. We asked if these cancer-associated mutations affect the interactions between the BRCT domains and pre-rRNA. We chose one of the most frequently found missense mutations in each BRCT domain (M1775R of the BRCA1 BRCT domain, C645R of the BARD1 BRCT domain)^21,34^. We generated recombinant mutant proteins and performed pull-down and RT-qPCR assays. Both mutations disrupted their interactions with pre-rRNA (Fig. 6a). We further measured the binding affinities between the mutant proteins and RNA oligos and confirmed that the interactions were largely abolished (Fig. 6b). Moreover, the mutant proteins could neither form LLPS with pre-rRNA in vitro nor localize onto DSBs in cells (Fig. 6c, d). In addition, these mutants failed to localize at the unsynapsed region in the XY body (Supplementary Fig. S12). Thus, these results suggest that cancer-associated missense mutations in the BRCT domains of *BRCA1* and *BARD1* abolish their interactions with pre-rRNA, which impairs the relocation of the BRCA1/BARD1 complex to DSBs.

### Pre-rRNA mediates BRCA1-dependent HR

**Fig. 7:**
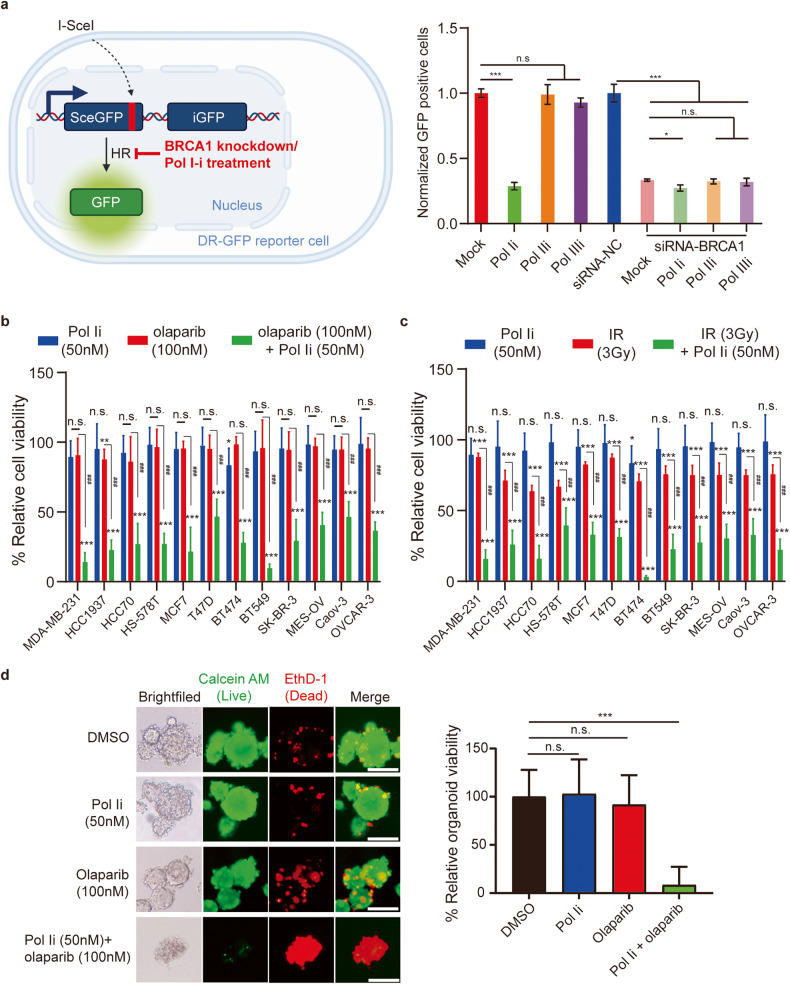
Pol-I inhibitor treatment sensitizes tumor cells to PARP inhibitor or IR treatment. **a**–**c** Pol Ii treatment impairs BRCA1-dependent HR repair. Cells were pre-treated with 1 μM of pol Ii, pol IIi or pol IIIi for 2 h. DR-GFP reporter assays were performed (**a**). Low-dose pol Ii (50 nM) treatment sensitizes the indicated breast and ovarian cancer cells to PARPi (olaparib, 100 nM) (**b**) or IR (3 Gy) (**c**). **d** Pol Ii (50 nM) and PARPi (olaparib 100 nM) synergistically suppresses TNBC organoids. *P* values were calculated using Student’s *t*-test. n.s. nonsignificant, *P < 0.05, ***P < 0.001. Scale bars, 100 μm.

Following the recruitment to DSBs, the BRCA1/BARD1 complex participates in HR repair. Using a well-established GFP reporter assay^35^, we examined the role of pre-rRNA in BRCA1-dependent HR. Since pre-rRNA is transcribed by pol I, we treated cells with pol-I inhibitor (pol Ii) to temporarily suppress pre-rRNA transcription, but not affect protein translation (Supplementary Fig. S13). Interestingly, similar to knockdown BRCA1 with siRNA, pol Ii treatment impaired HR repair (Fig. 7a and Supplementary Fig. S14), suggesting that pol-I-dependent pre-rRNA may play a key role in HR. As negative controls, neither pol II inhibitor nor pol III inhibitor treatment disrupted HR repair. Moreover, in the cells lacking BRCA1, the inhibitory effects of pol Ii were diminished (Fig. 7a), suggesting that pol-I-dependent pre-rRNA is epistatic with BRCA1 on HR.

Since pol Ii treatment impairs BRCA1-dependent HR, this treatment may cause BRCAness phenotype, which includes hypersensitivity to PARP inhibitor (PARPi) treatment^36^. With low-dose pol Ii treatment, a panel of breast and ovarian cells were still viable (Supplementary Fig. S15a). However, low-dose pol Ii treatment sensitized tumor cells to PARPi (Fig. 7b and Supplementary Fig. S15b), suggesting that pol Ii treatment may generate a BRCAness phenotype and acts as a sensitizer for PARPi treatment to suppress tumor cell growth. In addition, HR deficiency leads to cellular sensitivity to DNA-damaging agent treatment, such as IR. Thus, we examined and found that pol Ii treatment also sensitized tumor cells to IR treatment (Fig. 7c and Supplementary Fig. S15c).

Moreover, to examine the clinically relevant cancer samples, we cultured a 3D organoid that was derived from a triple-negative breast cancer (TNBC) patient. This TNBC organoid does not carry any *BRCA1* or *BRCA2* mutation (Supplementary Table S2), and it was sensitive to neither PARPi (IC_50_ was 8.23 μM) nor pol Ii treatment (Supplementary Fig. S16). Interestingly, this TNBC organoid was hypersensitive to the combination treatment of low-dose pol Ii (50 nM) and low-dose PARPi (100 nM) (Fig. 7d), suggesting that combination therapy using pol Ii and PARPi could be used for future cancer treatment.

## Discussion

In this study, we have shown that the BRCT domains of BRCA1 and BARD1 recognize pre-rRNA at DSBs. Both BRCT domains are known as phospho-protein-binding motifs^7^. However, none of the known binding partners target the BRCA1/BARD1 complex to DSBs. Here, we found that both BRCT domains bound to pre-rRNA, which mediates the recruitment of the BRCA1/BARD1 complex to DSBs. rRNA is often considered as a source of RNA contamination. Thus, the very first step of RNA sequencing analysis is to remove rRNA (aka “riboclear”)^37,38^. Here, we demonstrate that pre-rRNA is not RNA contamination, but localizes at DSBs. In particular, it localizes not only at genotoxic stress-induced DSBs in somatic cells, but also at physiologically relevant DSBs in the XY body of male germ cells. Moreover, we found that only RNase A treatment but not RNase H treatment could abolish the interactions between the BRCT domains and pre-rRNA (Fig. 1). Thus, it excludes the possibility of R-loops at rDNA loci mediating the foci formation of the BRCA1/BARD1 complex. Instead, pre-rRNA may act as a scaffold for the recruitment of the complex to DSBs. Moreover, these BRCT domains form LLPS with pre-rRNA. Since IRIF of the BRCA1/BARD1 complex is a unique type of phase separation at DSBs, these in vitro studies may provide the molecular basis of the foci formation of the BRCA1/BARD1 complex at DNA lesions. We understand that in the in vitro LLPS assays, the concentration of the BRCA1/BARD1 complex is beyond physiological levels. However, it is possible that in in vivo settings, local concentrations of the BRCA1/BARD1 complex and pre-rRNA may be high enough to form tiny protein–RNA droplets. Because of the quick fusion of these droplets, we are able to observe obvious IRIF at DSBs a few hours after genotoxic stress. The proposed foci fusion has also been reported by other groups^39,40^.

In addition, we observed that the RNase A treatment also affected the foci formation of γH2AX (Fig. 1d, e). Since the RNase A treatment was conducted before cell fixation, thus abolishing the RNA scaffold for the foci formation. In our earlier studies, we have shown that MDC1 recognizes and loads pre-rRNA to DSBs via the interactions between FHA-PST of MDC1 and pre-rRNA^32^. Meanwhile, the BRCT domain of MDC1 is known to recognize pSer139 motif of H2AX^41,42^. Thus, pre-rRNA likely acts as the scaffold to maintain the IRIF at DSBs. Upon RNase A treatment, this RNA scaffold is disrupted, IRIF is impaired, and we found that γH2AX foci became fuzzy and vague (Supplementary Fig. S17). Consistently, our previous studies have also shown that pol Ii treatment suppresses pre-rRNA biogenesis and impairs γH2AX foci formation^32^.

Both BRCT domains recognize phosphate moieties. The BRCA1 BRCT domain is known to recognize a phospho-Ser motif with +3 position as a Phe^7,9^. The phosphate-binding pocket is composed of Ser1655 and Lys1702, which catch the phosphate group as chopsticks. In addition, the side chains of F1704, M1775 and L1839 form a hydrophobic pocket to host the side chain of +3 Phe^34^. Interestingly, pre-rRNA also contains large amount of phosphate groups. It is likely that the phosphate-binding pocket of BRCT recognizes a phosphate moiety in RNA. Moreover, both purine and pyrimidine contain similar ring structure. Thus, it is likely that the hydrophobic pocket holds the ring structure of purine or pyrimidine from RNA. Slightly different from BRCA1 BRCT domain, the BARD1 BRCT domain recognize phosphor-Ser with +1 position as an acidic residue^9^. Since phosphate groups contain negative charges, it is possible that the BARD1 BRCT domain recognizes tandem phosphate groups in pre-rRNA. Currently, we do not know if specific RNA sequences or secondary structures of pre-rRNA are recognized by the BRCA1/BARD1 complex. Future structural analyses will be needed to reveal the detailed binding mode between the BRCT domains and pre-rRNA. Moreover, recent studies indicate that the BARD1 BRCT domain is a ubiquitin-binding motif^43–45^. It is possible that the BRCT domain of BARD1 uses different surface areas to interact with ubiquitin for a different biological process. In addition to the BRCT domain, other motifs of the BRCA1 BARD1 complex may also interact with pre-rRNA. Usually, the protein–RNA complex contains multivalent interactions^46^. Thus, future studies on this complex may reveal additional RNA-binding motifs.

In the BRCT domains, there are numerous cancer-associated mutations. Some of them abolish the tertiary structure of the BRCT domains, while others impair the binding of their partners^34^. Here, we found that M1775R disrupted the interaction between the BRCA1 BRCT domain and pre-rRNA. The side chains of M1775R extends toward the surface of the BRCT domain and contributes to an important hydrophobic pocket that may host pre-rRNA^34^. In addition, the C645R mutant of the BARD1 BRCT domain likely destabilizes the first BRCT repeat^21^. Thus, we observed that these mutations abolish the interactions between the BRCT domains and pre-rRNA, and impair the pre-rRNA-mediated relocation of BRCA1 and BARD1 to DNA lesions.

In addition to the DNA-damaging agent-induced foci, the BRCA1/BARD1 complex is also recruited to the unsynapsed region in the XY body, which is also occupied by other HR repair machinery as well as pre-rRNA^32,47^. Our earlier studies have shown that the XY body is the biggest DSB repair center under light microscopy, in which both X and Y chromosomes are cut by SPO11^28,47,48^. Due to the lack of homology, DSB repair on the X and Y chromosomes is delayed. Thus, X and Y chromosome conjugate together to form LLPS^28^, known as the XY body. Since we demonstrate that the BRCT domains recognize pre-rRNA, it is likely that pre-rRNA mediates the recruitment of the BRCA1/BARD1 complex to not only IRIF but also the unsynapsed region in the XY body for HR repair. In addition to the BRCA1/BARD1 complex, pre-rRNA, a putative scaffold RNA at DNA lesions, may also mediate the recruitment of other DSB repair factors^32^. It is possible that different repair factors recognize different portions of pre-rRNA. Systematical analyses on different DSB factors are needed to elucidate the biological functions of pre-rRNA in DSB repair. But it is beyond the scope of this study. Nevertheless, current studies clearly reveal the role of pre-rRNA in recruiting the BRCA1/BARD1 complex to DSBs also in facilitating the BRCA1-dependent HR repair, which opens a new avenue for reanalyzing other known DSB repair factors.

In addition to pre-rRNA, the BRCA1/BARD1 complex also interacts with pre-rRNA-associated proteins, which are also revealed as DSB repair factors by other groups^49^. Thus, it is possible that these pre-rRNA-associated proteins carry out functions in DSB repair as well. Further analyses on each pre-rRNA-associated protein may uncover novel networks of DSB repair pathways in the future.

In addition to pol-I-mediated DNA damage repair, BRCA1 is also known to regulate the activity of pol I and pre-rRNA biogenesis^50^. BRCA1 interacts directly with pol-I transcription machinery, and promotes pre-rRNA synthesis in cells^50^. In addition to pre-rRNA, BRCA1 also regulates the transcription of ribosomal proteins^51,52^. Here we found that the BRCA1/BARD1 complex binds to a large number of ribosomal proteins and pre-rRNAs. Thus, it is possible that BRCA1 and pol I may form a feedback loop, which could be a new layer of regulation for DSB repair.

In somatic cells, we have shown that pol I, the enzyme for pre-rRNA transcription, is required for HR repair. Currently, two different types of pol-I inhibitors are in clinical trials for the treatments of various types of cancers. Our study demonstrates that pol Ii treatment sensitizes tumor cells to PARPi treatment. Thus, it is possible that pol-I inhibitors treatment may be utilized together with PARPi for cancer treatment in the future. Collectively, our results demonstrate that pol-I-mediated pre-rRNA biogenesis plays a key role for HR repair by recruiting the BRCA1/BARD1 complex to DSBs.

## Materials and methods

### Study approval

All mouse experiments were permitted by Westlake University Animal Care and Use Committee and were performed according to the approved protocol (20-029-YXC). Mice were obtained from Westlake University Animal Center. The human breast cancer specimen was collected in accordance with the Declaration of Helsinki and was approved by the Clinical Research Ethics Committee of the First Affiliated Hospital, Zhejiang University School of Medicine (No. IIT20210161B). Informed consent was obtained from the participant.

### Cell line and reagents

HeLa and 293T cells were purchased from American Type Culture Collection. Cells were cultured in DMEM (Thermo Fisher Scientific) containing 10% fetal bovine serum and 1% penicillin–streptomycin (Invitrogen) at 37 °C with 5% CO_2_.

To generate 293T cells stably expressing BRCA1 BRCT domain or full-length BARD1, SFB-Puro vectors containing target DNA fragments were transfected into 293T cells using Lipofectamine 2000 (Thermo Fisher Scientific) according to the manufacturer’s protocol. The transfected cells were selected in a medium containing 2 μg/mL puromycin. The sequence of BRCA1 siRNA oligonucleotides was 5’-AAGGUUUCAAAGCGCCAGUCA-3’. The control siRNA was a scrambled sequence. Cells were transfected with siRNA using Lipofectamine RNAiMAX (Thermo Fisher Scientific).

Anti-γH2AX (NB100-384) and anti-SCP3 (NB300-232) antibodies were purchased from Novus. Anti-FLAG (8146S) and anti-GST (2624S) antibodies were purchased from Cell Signaling Technology. Anti-RPL7A (A13713) and anti-RPS3 (A2533) antibodies were purchased from ABclonal. Anti-RPL14 (14991-1-AP) antibodies were purchased from Proteintech. Anti-BRCA1 (MS110) was purchased from Sigma. Anti-BARD1 antibody was purchased from Novus. Anti-Rad51 antibody was purchased from Abcam (ab133534). BMH-21(S7718), α-amanitin (A4548) and ML-60218 (557403) were purchased from Selleck, APExBIO, and Sigma, respectively.

### Plasmid constructs

The cDNA encoding the BRCA1 BRCT domain or full-length BARD1 was cloned into SFB vector. To generate GST-tagged protein, DNA fragment encoding BRCA1 BRCT or BARD1 BRCT was cloned into pGEX-4T1 vector. GST-tagged proteins were expressed in BL21 bacteria, purified using glutathione sepharose (GE) and SuperDex 200 (Cytiva) with Fast Protein Liquid Chromatography (FPLC, Union Bio). Point mutations were introduced by Quick change site-directed mutagenesis and confirmed by DNA sequencing. To express BRCTs in MDA-MB-231 and MDA-MB-436 cells, BRCA1 BRCT (a.a. 1589–1863) or BARD1 BRCT (a.a. 559–777) with a nuclear localization signal was subcloned into SFB vector.

### Immunofluorescence (IF) staining

Cells were cultured on coverslips and treated with or without 10 Gy of IR. After 12-h recovery, cells were fixed with 4% paraformaldehyde (PFA) for 15 min, and treated with 0.5% Triton X-100 in PBS for 5 min. The coverslips were incubated with primary antibodies for 1 h. For GST fusion protein binding, the coverslips were incubated with the proteins or protein/RNA mixture for 1 h before primary antibody incubation. Then, the coverslips were washed three times with PBS and incubated with secondary antibodies for 1 h. The coverslips were subsequently washed three times with PBS and mounted. All images were taken using Olympus FV3000 confocal microscope.

For RNase A/H treatment, cells were treated with 1 μg/mL RNase for 30 min at 37 °C after 0.5% Triton X-100 treatment, then fixed. For RNA polymerase inhibitor treatments, 1 μM pol Ii (BMH-21, Selleck CAS S7718), pol IIi (α-Amanitin, R&D system CAS 4025), or pol IIIi (RNA Polymerase III Inhibitor, CAS 577784-91-9) was added into cell culture media, respectively, and treated for 2 h before IR.

To examine the foci of BRCTs, cells expressing SFB–BRCA1 BRCT or SFB–BARD1 BRCT were treated with 10 Gy of IR. IF was performed with the indicated antibodies.

### Co-IP, mass spectrometry, and western blotting assay

293T cells stably expressing BRCA1 BRCT or BARD1 were lysed by NETN buffer. The cell lysates were centrifuged at 13,000 rpm at 4 °C for 30 min. Streptavidin agarose resin (Thermo Fisher Scientific, 20353) was added into supernatant for incubation at 4 °C for 2 h. After washing the resin with NETN100 buffer for three times, associated proteins were eluted with 2 mg/mL biotin. The second pull-down was performed with anti-FLAG® M2 affinity gel (Sigma, A2220). The eluded proteins were sent for analysis at the mass spectrometry core facility of Westlake University.

For endogenous co-IP assay, 293T cells were lysed with NETN buffer. The cell lysates were incubated with anti-BRCA1 or BARD1 antibody for 1 h at 4 °C. For phosphatase treatment, the IPed samples were treated with 20 unit/mL l phosphatase for 30 min at 37 °C. For RNA depletion, the IPed samples were treated with 1 mg/mL RNase A for 30 min at 37 °C. The IPed samples were boiled in SDS sample buffer. For western blotting assay, the samples were examined with indicated antibodies.

### Surface spread of spermatocytes

Testis was decapsulated, and seminiferous tubules were then incubated in HEB buffer (30 mM Tris-HCl, pH 8.2, 50 mM sucrose, 17 mM sodium citrate, 5 mM EDTA, 0.5 mM dithiothreitol and 0.1 mM phenylmethylsulfonyl fluoride) for 30 min at room temperature. The same volume of 100 mM sucrose was added to the isolated spermatocytes in the HEB buffer. The cells were then filtered by a cell strainer before being spread onto slides pre-coated with fixation buffer (1% PFA, 0.15% Triton X-100, sodium borate, pH 9.2). The slides were kept in humidified chambers for 2 h, and air-dried.

### IF staining and RNA FISH (fluorescence in situ hybridization) for spermatocytes

Slides were incubated with primary antibodies diluted with 1% BSA for 1 h at room temperature, followed by incubation with corresponding secondary antibodies for 30 min at room temperature. Of note, the IF staining was performed after RNA FISH staining for the detection of both RNAs and proteins.

For RNA FISH, meiotic spreads were first incubated with wash buffer A (SMF-WA1-60, Bioresearch Technologies, USA) for 5 min at room temperature. Slides were then incubated with probes diluted by hybridization buffer (SMF-HB1-10, Bioresearch technologies, USA) overnight at 37 °C. After incubation, the slides were washed by wash buffer A for 10–30 min at room temperature. Probes are listed in Supplementary Table S3.

### RNA and GST-protein pull-down assay

Purified N-terminal GST-tagged proteins were incubated with total RNA and Glutathione Sepharose^TM^ 4B (17-0756-05, GE Healthcare) at 4 °C for 2 h. The reaction mixture was washed by NETN100 three times at 4 °C, and the supernatant was discarded. RNA was extracted with TRIzol reagent (15596026, Thermo Fisher). Finally, the protein-binding RNA was purified by centrifugation (13,000 rpm at 4 °C for 10 min). RT-qPCR was performed to detect the purified RNA. The primers are listed in Supplementary Table S3.

### Incubation of recombinant proteins with meiotic spreads and irradiated cells

GST fusion proteins were incubated with meiotic spreads for 1 h at 37 °C. Anti-GST antibodies were used in IF staining subsequently. For phosphatase treatment, meiotic spreads were treated with 20 unit/mL l phosphatase for 30 min at 37 °C. For RNA depletion, meiotic spreads were treated with 1 mg/mL RNase A for 30 min at 37 °C. For RNA blocking, GST fusion proteins were pre-incubated with excessive pre-rRNA.

### Pre-rRNA preparation

Cells were incubated with permeabilization buffer (10 mM HEPES, pH 7.4, 10 mM KCl, 0.05% NP-40) on ice for 10 min. Samples were centrifuged at 5000× *g* for 5 min at 4 °C. The pellets were harvested and washed again with permeabilization buffer, and centrifuged at 5000× *g* for 5 min at 4 °C. Supernatants were discarded and pellets were dispersed with CSK (10 mM PIPES, pH 7.0, 100 mM NaCl, 300 mM sucrose, 3 mM MgCl_2_) buffer on ice for 10 min and centrifuged at 5000× *g* for 5 min at 4 °C. The remaining pellets were washed with CSK buffer and centrifuged at 5000× *g* for 5 min at 4 °C. Supernatants were discarded, and pre-rRNA was extracted from the pellets using TRIzol.

### Biolayer interferometry (BLI)

The interactions between the BRCTs and RNA were determined using Octet Red96e (Sartorius). 5’-biotinylated RNA oligo was synthesized by Tsingke Biotechnology Co., Ltd. For binding affinity analysis, biotinylated RNA was captured on streptavidin biosensors. Biotinylated RNA was diluted to 500 ng/mL in kinetics buffer (PBS with 0.05% Tween-20). The BRCTs were diluted to different concentrations in kinetics buffer. The sensor baselines were equilibrated in kinetics buffer for 120 s. Next, biotinylated RNA was loaded until saturation. Then, the sensors were washed in kinetics buffer, and were immersed in wells containing BRCTs (association phase), followed by immersion in kinetics buffer (dissociation phase). The background signals were measured using a reference sensor with biotinylated RNA oligo but without analytes and was subtracted from the corresponding BRCT-binding sensor. The mean *K*on, *K*off, and *K*D values were calculated with Octet Data Analysis HT 12.0 software using a 1:1 global fit model, and the theoretical fit with *R*^2^ > 0.95. Data were plotted using GraphPad Prism V8.0 software (GraphPad).

### In vitro phase separation assay

For the in vitro LLPS experiments, recombinant BRCTs were mixed with pre-rRNA in the phase separation buffer (100 mM NaCl, 50 mM Tris-HCl, pH 7.4, and 10% PEG 8000 (NEB)) and were directly pipetted onto a coverslip under the microscope. During the LLPS experiments, every dynamic process was observed by an Olympus microscope with 40× differential interference contrast.

### PAR-CLIP library preparation and sequencing

293T cells stably expressing BRCA1 BRCT or BARD1 were incubated with 0.1 mM 6SG overnight, followed by 0.4 J cm^2^ 365 nm wavelength UV treatment. Cells were harvested and lysed by NETN100 buffer. The cell lysates were centrifuged at 13,000 rpm at 4 °C for 30 min. Streptavidin agarose resins (Thermo Fisher Scientific, 20353) were added into the supernatant for incubation at 4 °C for 2 h. The resins were washed with NETN300 buffer three times, 2 M urea once, and NETN100 buffer twice, respectively. RNA–protein-binding complexes were eluted with 2 mg/mL biotin. The second pull-down was performed with anti-FLAG® M2 beads (Sigma, A2220). The beads were washed with NETN300 buffer three times, 2 M urea once, and NETN100 buffer twice, respectively. Proteinase K was added to digest proteins, and TRIzol was used to extract binding RNAs.

The RNA samples were examined by Qubit 3.0 Fluorometer (Invitrogen, Carlsbad, California), and Agilent 2100 Bioanalyzer (Applied Biosystems, Carlsbad, CA), respectively. RNA-seq library was prepared with ~10 ng of total RNA using Clontech SMARTer Stranded Total RNA-seq kit V2 pico input (Takara). The library quality and concentration were assessed by utilizing a DNA 1000 chip on an Agilent 2100 Bioanalyzer. Each library was diluted to a final concentration of 10 nM and pooled equimolar before clustering. Illumina Novaseq 6000 PE150 sequencing was performed on all samples.

### RNA sequencing analysis

Human T2T-CHM13 v1.1 reference sequence and annotation of file were used in STAR alignment, and 45 S sequence of the human KY962518.1 reference genome was obtained from NCBI Nucleotide database.

The raw Fastq files were analyzed using FastQC and cleaned. Cleaned Fastq files were aligned to the human T2T-CHM13 v1.1 reference genome using STAR (v2.7.8a), and the unaligned reads in this step were aligned to KY962518.1 45 S sequence using BLASTN (v2.11.0+). The expression level of genes in PAR-CLIP sequencing data was quantified by FeatureCounts (v2.0.1). Normalized counts of 45 S were quantified in each 10 bp bin. The rRNA biotype fractions were calculated by the ‘biotype’ annotation with the addition of BLASTN-aligned read counts.

### Protein synthesis measurement

The protein synthesis of cells treated with vehicle (DMSO) or pol Ii (BMH-21) was examined by the incorporation of puromycin into peptide chains. In brief, cells were first treated with DMSO or BMH-21 (1 μM) and subsequently incorporated with puromycin for 10 min. Then the cells were collected for western blotting. An anti-Puromycin antibody (clone 12D10, Merk, MABE343) was applied to quantitate the overall neosynthesized protein.

### Detection of homologous recombination repair

For the homologous recombination repair assays, we have generated an inducible I-SceI system^10^. HA-I-SceI-GR and pCW tet-on plasmids were transfected to the cells. Doxycycline was used to induce I-SceI expression. Cells were treated with RNA polymerase I, II, III inhibitor for 2 h, respectively, rinsed and replaced with fresh media. Then acetonide (TA) was applied to induce the translation of I-SceI from cytoplasm to nucleus, which induced DSB at the I-SceI sites. Once this DSB was repaired, the GFP expressed. After TA was treated for 10 h, flow cytometry was performed to examine the efficiency of HR repair.

### Detection of pre-rRNA and its associated proteins in IRIF

Cells were plated on glass coverslips and treated with 10 Gy of IR. The cells were treated with HEB buffer for 30 min and then followed with sucrose solution (100 mM) for 30 min. The cells were fixed with 2% PFA and permeabilized with 0.2% Triton X-100 in PBS for 30 min at room temperature. Stellaris RNA FISH and IF were performed according to the manufacturer’s protocol. Pre-rRNA probes are listed in Supplementary Table S3.

### Cell viability assay

A total of 2 × 10^3^ cells were seeded in each well of 96-well plates. After 24 h, cells were treated with the indicated concentration of pol-I inhibitor or olaparib, the medium was changed every day. After incubation for 6 days, the surviving cells were assessed with CellTiter-Glo reagent (Promega) following the manufacturer’s instructions.

### 3D organoid culture

Inflammatory breast cancer cells were used for the 3D in vitro assay. Cells were suspended in M3A medium to achieve a concentration of 1 × 10^5^ cells/mL. 100 µL of the single-cell suspension was added to each well of a 96-well plate that was pre-coated with Matrigel (Corning, 356231). The cells were incubated at 37 °C in a 5% CO_2_ humidified incubator overnight. For olaparib or pol Ii treatment, compounds were diluted in M3A medium to 2× the desired final concentration. DMSO treatment was used as a control. 100 µL of M3A medium containing 2× compound or DMSO was added to each well. Drug contained medium was replaced for every two days. The cells were maintained for 7 days.

### Organoid viability assay

To detect the viability of organoids, Viability/Cytotoxicity kit (calcein AM/ethidium bromide homodimer-1) (Thermofish, L3224) was used following the manufacturer’s instructions. Images of calcein AM (ex/em 494/517 nm) fluorescence, which represents the live cells, were captured. In addition, images of ethidium bromide (ex/em 528/617 nm) were acquired to identify dead cells. Fiji was used to analyze the fluorescence intensity. Each experiment was repeated three times.

### Statistical analysis

Data were analyzed with GraphPad Prism 8.0 software. The data presented were means ± SD. Statistical significance between the two groups was subjected to Student’s *t*-test. *P* < 0.05 was considered statistically significant.

### Supplementary information

supplementary table s2

supplementary information

supplementary table s1

## Data Availability

All datasets have been deposited in the GEO datasets under the GEO accession number GSE207338.
