# Supplementary material for: The BRCA1/BARD1 complex recognizes pre-ribosomal RNA to facilitate homologous recombination
Source: Cell Discov. 2023 Oct 3;9:99. doi: 10.1038/s41421-023-00590-8 (PMC10547766; doi:10.1038/s41421-023-00590-8)
Supplement: Supplementary file 2 — supplementary information [file 41421_2023_590_MOESM2_ESM.pdf]

Supplementary Figure

a

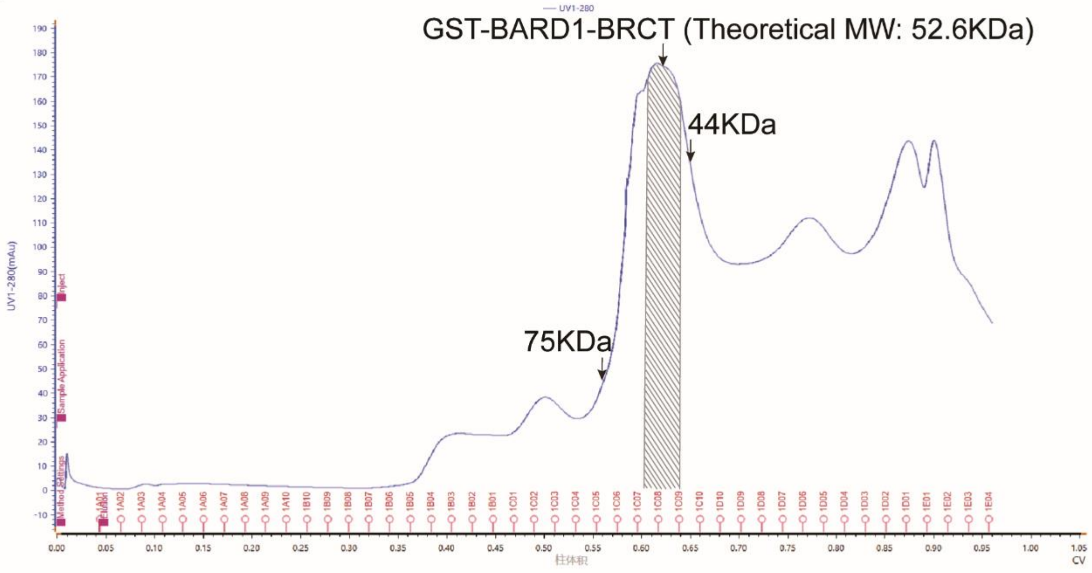

b

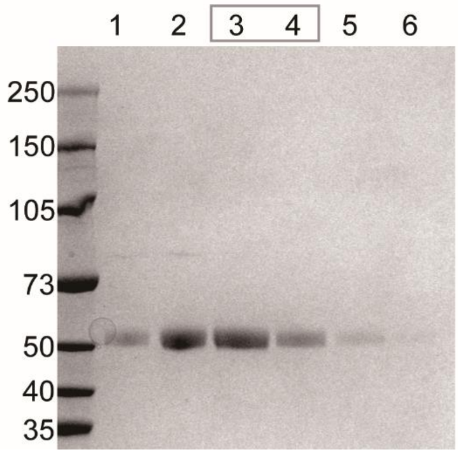

Supplementary Fig. S1 Obtained monomer form of GST-tagged BARD1 BRCT protein.

(a) Fast Protein Liquid Chromatography with SuperDex 200 was used to purify the monomer of GST-BARD1-BRCT protein. The gray shaded area indicates the elution fractions of the monomer of GST-BARD1-BRCT. **(b)** The elution fractions were examined by Western blot. Lane 3-4 corresponds to the gray-shaded fractions.

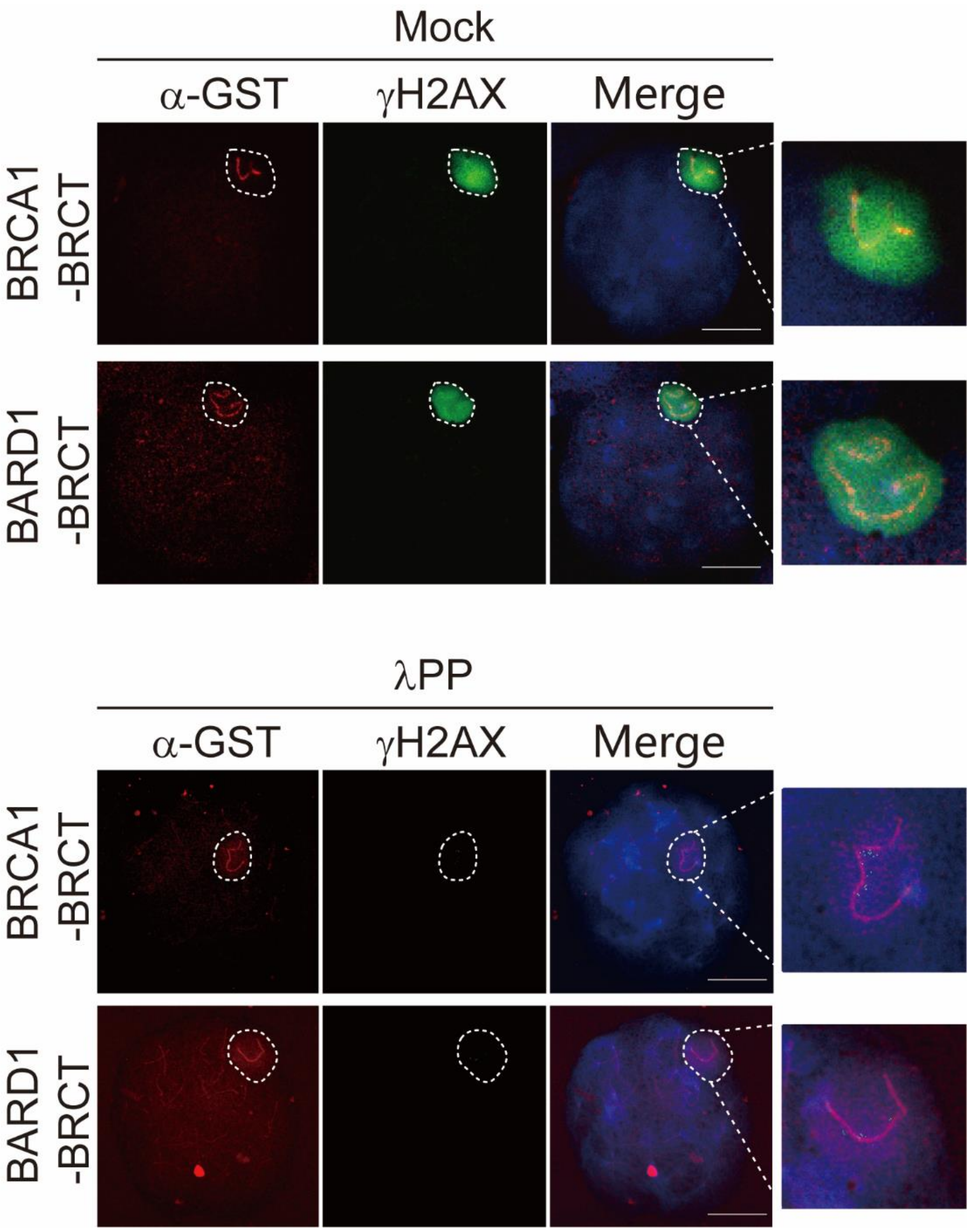

Supplementary Fig. S2 The binding of the recombinant BRCA1 BRCT or BARD1 BRCT to the XY body is not affected by λ phosphatase treatment.

Meiotic spreads were pretreated with or without λ phosphatase before incubation with the BRCTs. γH2AX is a surrogate marker for the XY body. Loss of γH2AX signal served as the positive control for the λ phosphatase treatment.

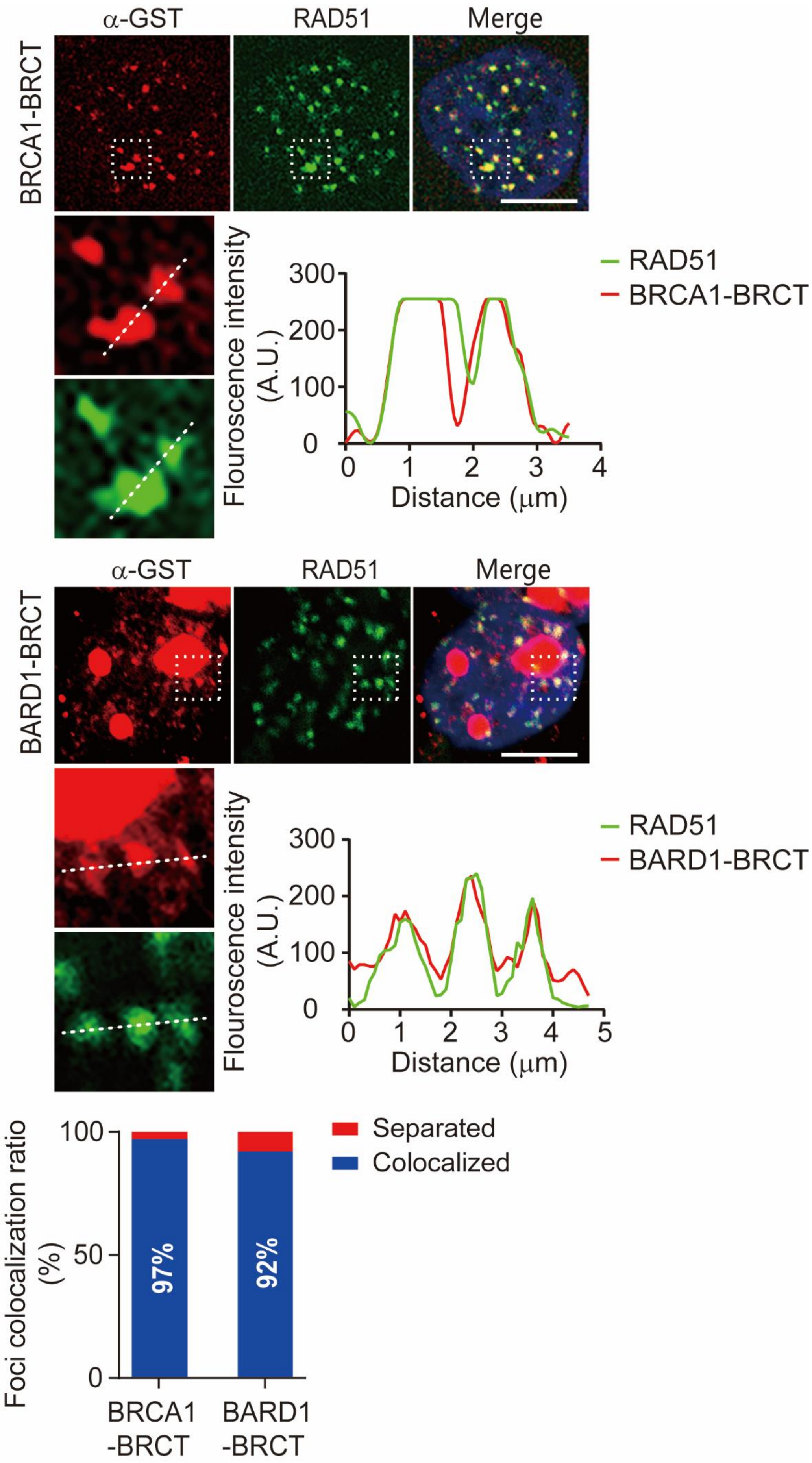
α-GST
RAD51
Merge
BRCA1-BRCT
Flouroscence intensity (A.U.)
RAD51
BRCA1-BRCT
Distance (μm)
α-GST
RAD51
Merge
BARD1-BRCT
Flouroscence intensity (A.U.)
RAD51
BARD1-BRCT
Distance (μm)
Foci colocalization ratio (%)
Separated
Colocalized
97%
92%
BRCA1 -BRCT
BARD1 -BRCT

Supplementary Fig. S3 The foci recombinant BRCA1 BRCT or BARD1 BRCT localize with the foci of RAD51 in the IR-treated U2OS cells.

U2OS cells were treated with 10 Gy of IR. After 12-hour recovery, the cells were incubated with the recombinant BRCA1 BRCT or BARD1 BRCT. Recombinant proteins were labeled with anti-GST antibody. The cells were co-stained with anti-RAD51 antibody. The fluorescence signals on the dash lines were analyzed. Foci colocalization ratio is examined.

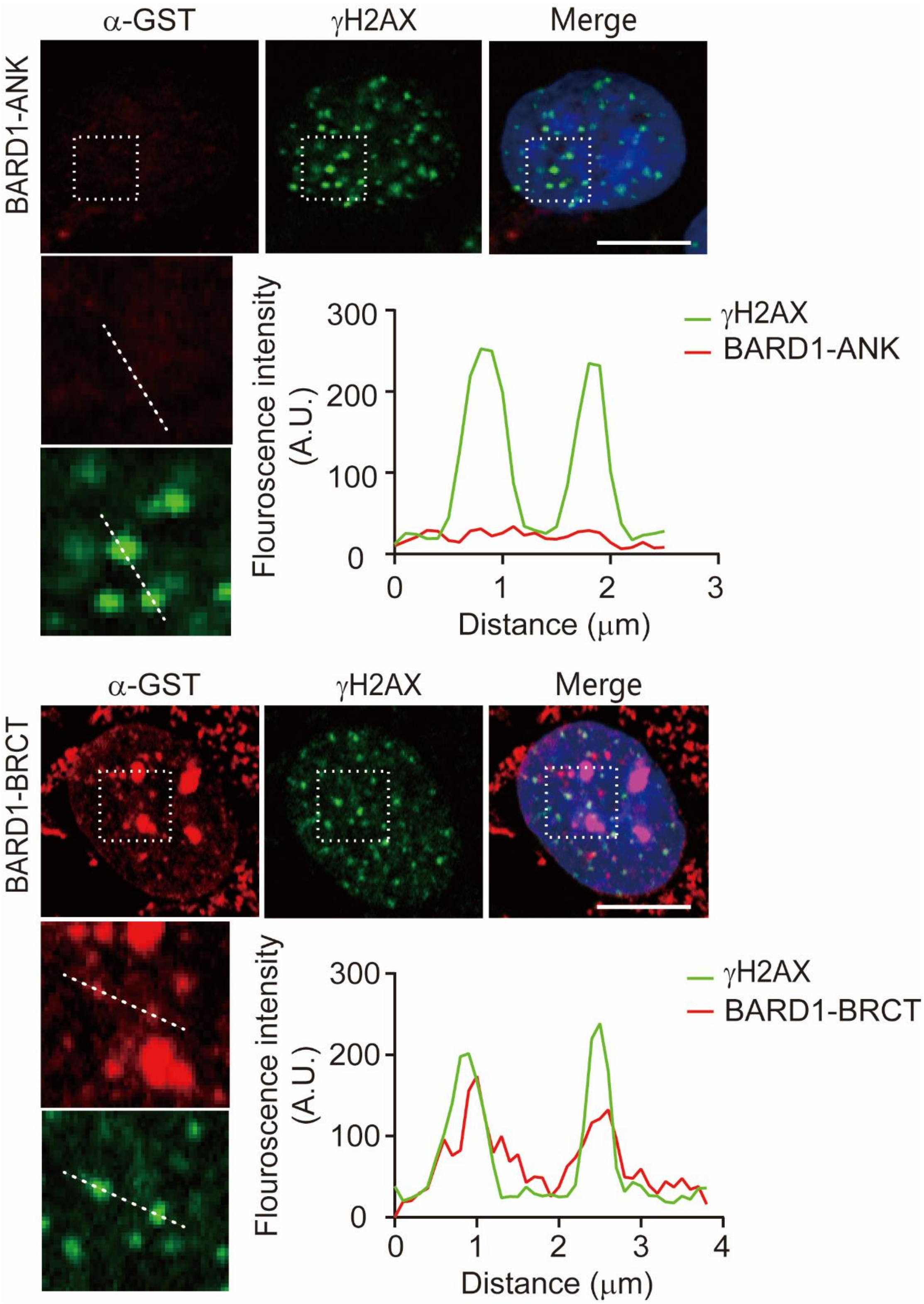

Supplementary Fig. S4 Incubation recombinant BARD1 ANK or BRCT with IR-treated U2OS cells.

U2OS cells were treated with 10 Gy of IR. After a 12-hour recovery, the cells were incubated with the recombinant BARD1-ANK or BARD1-BRCT. Recombinant proteins were labeled with anti-GST antibody, and DSBs were labeled with anti-γH2AX antibody. The colocalization fluorescence signals were analyzed.

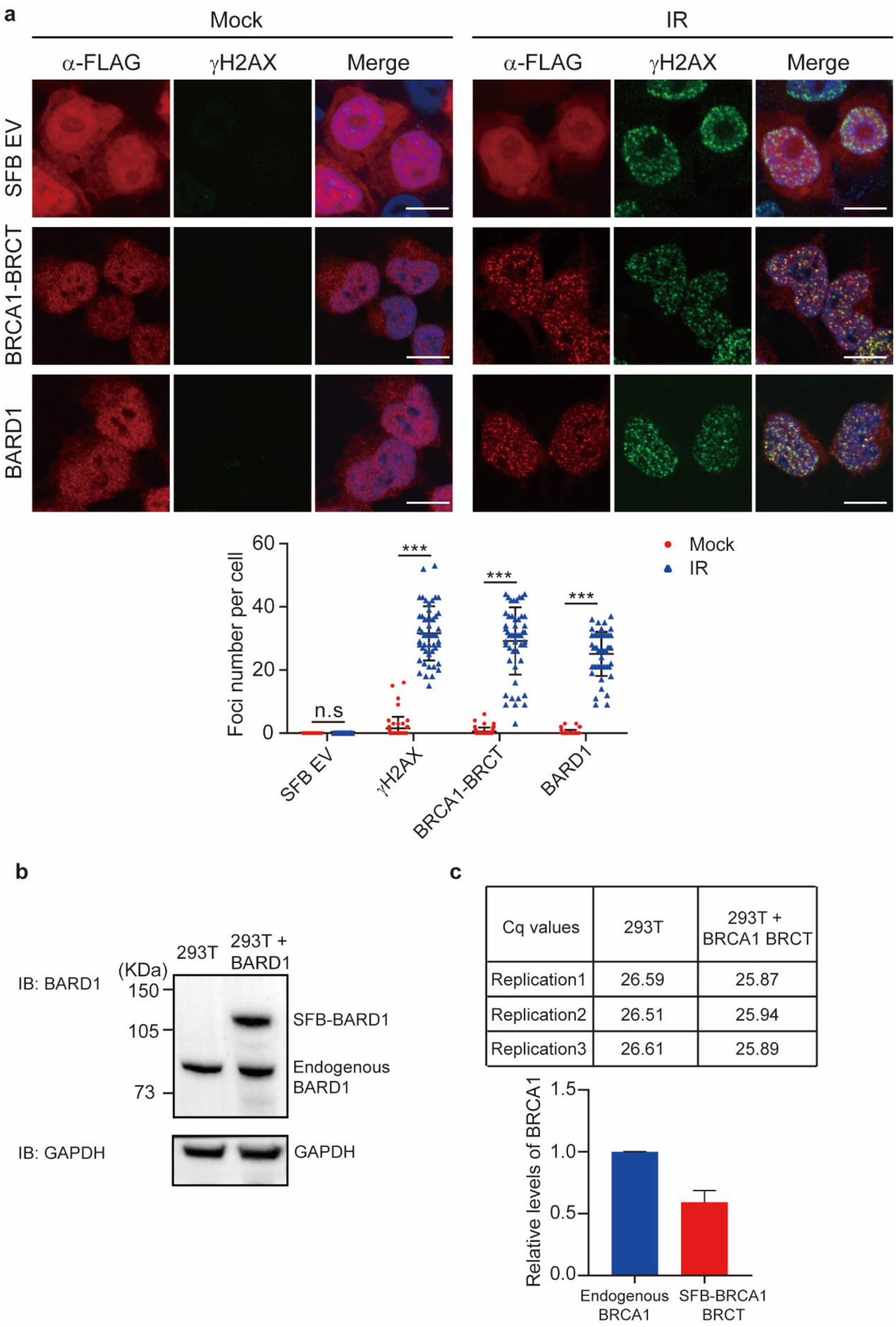

Cq values	293T	293T + BRCA1 BRCT
Replication1	26.59	25.87
Replication2	26.51	25.94
Replication3	26.61	25.89

Supplementary Fig. S5 Ectopically expressed BARD1 or the BRCA1 BRCT domain forms IRIF.

(a) 293T cells stably expressing SFB-BRCA1 BRCT or SFB-BARD1 were treated with 10 Gy of IR. IR-induced foci were examined with anti-FLAG antibody, γH2AX serves as a DSB marker. The foci number per cell was analyzed and shown in the bottom panel. P-values were calculated using Student's t-test. n.s.: nonsignificant, *P < 0.05, and ***P < 0.001. The image bar is 10 μm. **(b)** The expression of BARD1 was examined by Western blot with an anti-BARD1 antibody. **(c)** The expression of endogenous BRCA1 and ectopically expressed BRCA1 BRCT was examined by RT-qPCR. The Cq values are shown. The relative expression of endogenous BRCA1 and exogenous BRCA1 BRCT was calculated and compared in the histogram. The expression of GAPDH was used as the loading control for RT-qPCR. The primers used were shown in Supplementary Table S3.

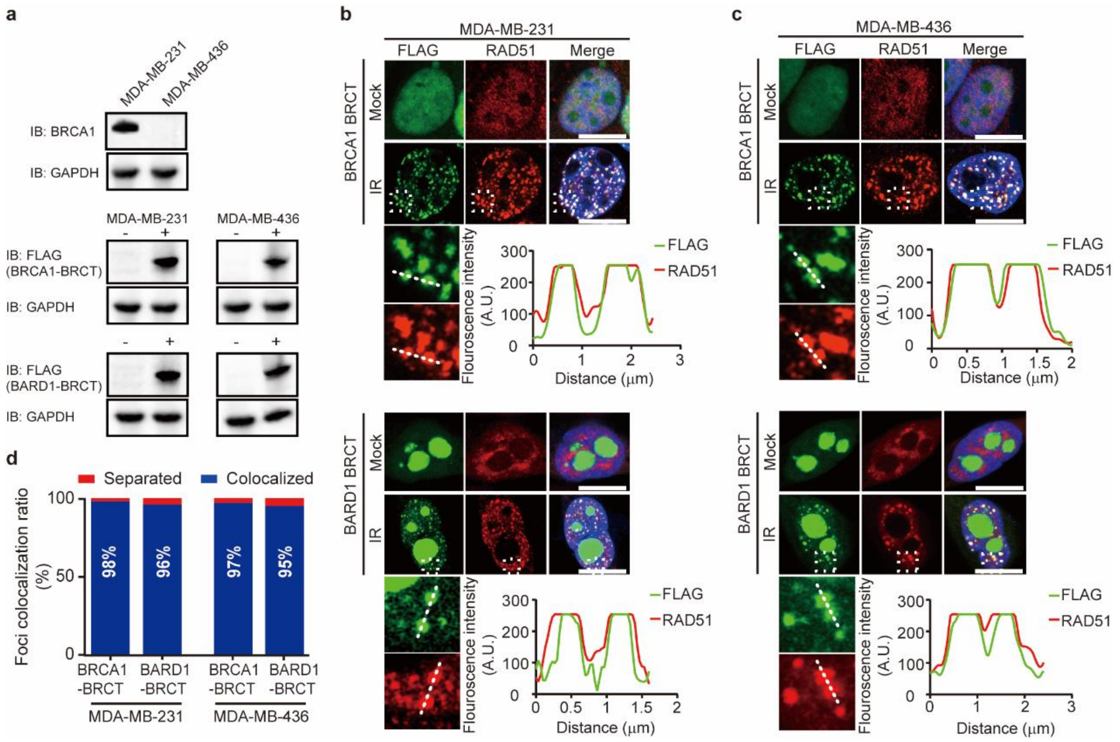

Supplementary Fig. S6 Ectopically expressed BRCA1 BRCT or BARD1 BRCT domain colocalizes with RAD51.

(a) SFB-BRCA1 BRCT or SFB-BARD1 BRCT was expressed in MDA-MB-231 or MDA-MB-436 cells. Anti-FLAG antibodies were used to detect the ectopically expressed BRCTs. The endogenous BRCA1 was examined by Western blot using anti-BRCA1 antibody. **(b and c)** The cells expressing the BRCTs were treated with 10 Gy of IR. IR-induced foci were examined with anti-FLAG antibody, RAD51 serves as a DSB marker. The image bar is 10 μm. **(d)** Co-localization of BRCT foci and RAD51 foci was analyzed.

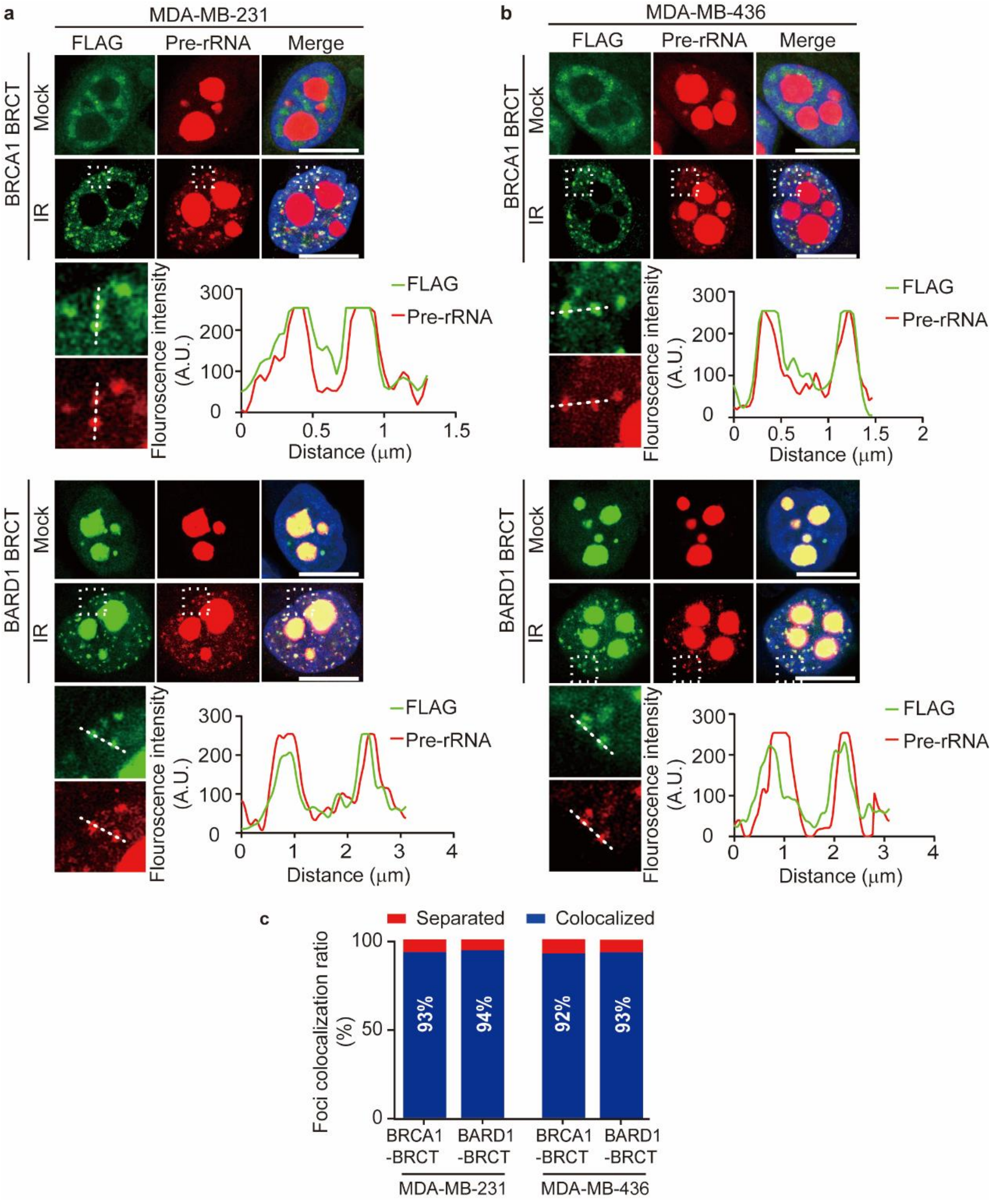

Supplementary Fig. S7 Ectopically expressed BARD1 or the BRCA1 BRCT domain colocalizes with pre-rRNA.

Colocalization of the BRCT foci and pre-rRNA foci was examined in MDA-MB-231 **(a)** or MDA-MB-436 **(b)** cells. The image bar is 10 μm. **(c)** The ratio of the foci colocalization was analyzed.

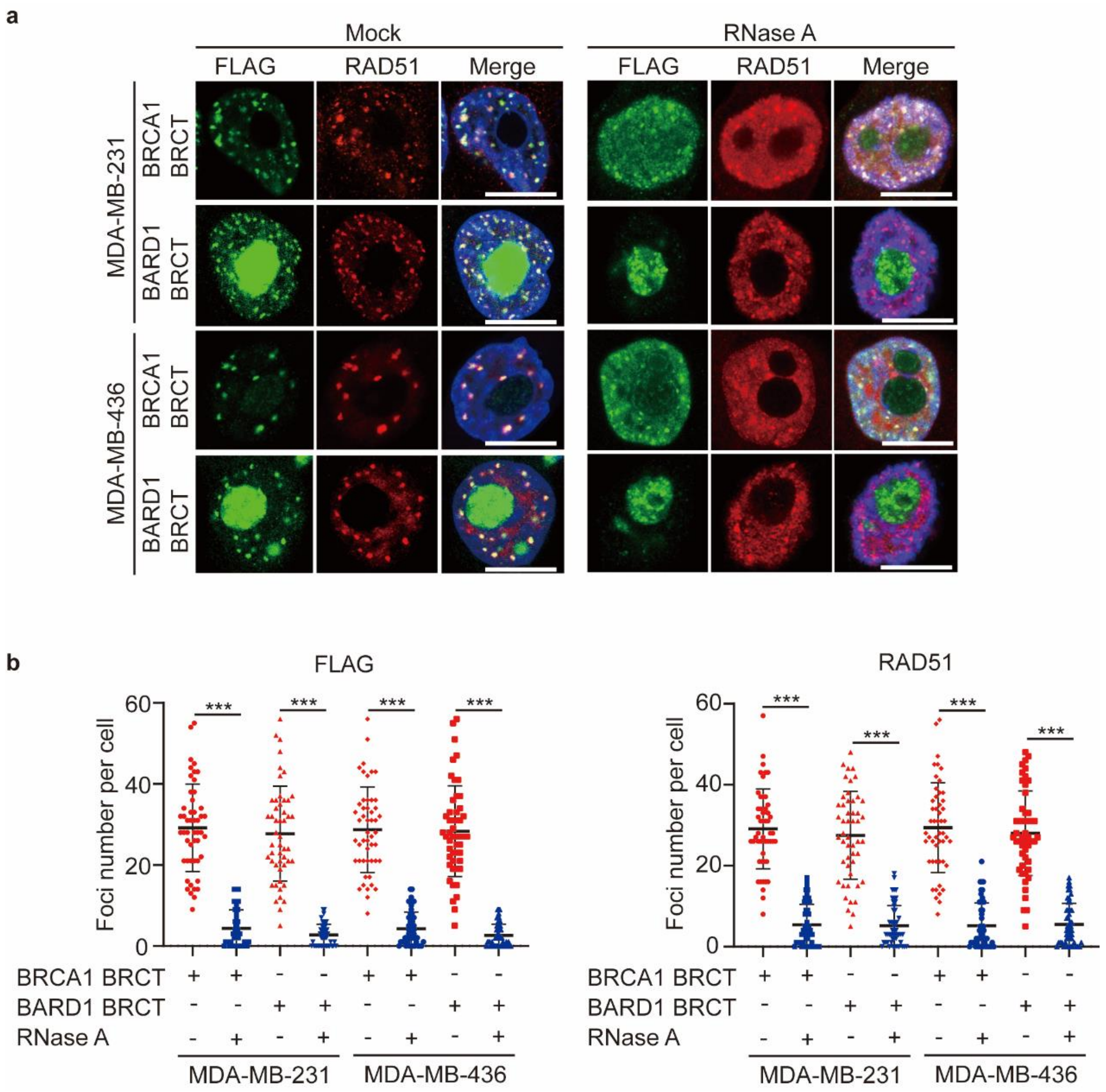

Supplementary Fig. S8 RNase A treatment abolishes the foci formation of BRCA1 BRCT or BARD1 BRCT.

(a) The cells expressing the BRCTs were treated with 10 Gy of IR. IR-induced foci were examined with anti-FLAG antibody and anti-RAD51 antibody. For the RNase A treatment, cells were pre-treated with 1 μg/ml RNase A for 30 min before fixation. The image bar is 10 μm. **(b)** Foci number per cell was examined. P-values were calculated using Student's t-test. n.s.: nonsignificant, $*P < 0.05$, and $***P < 0.001$.

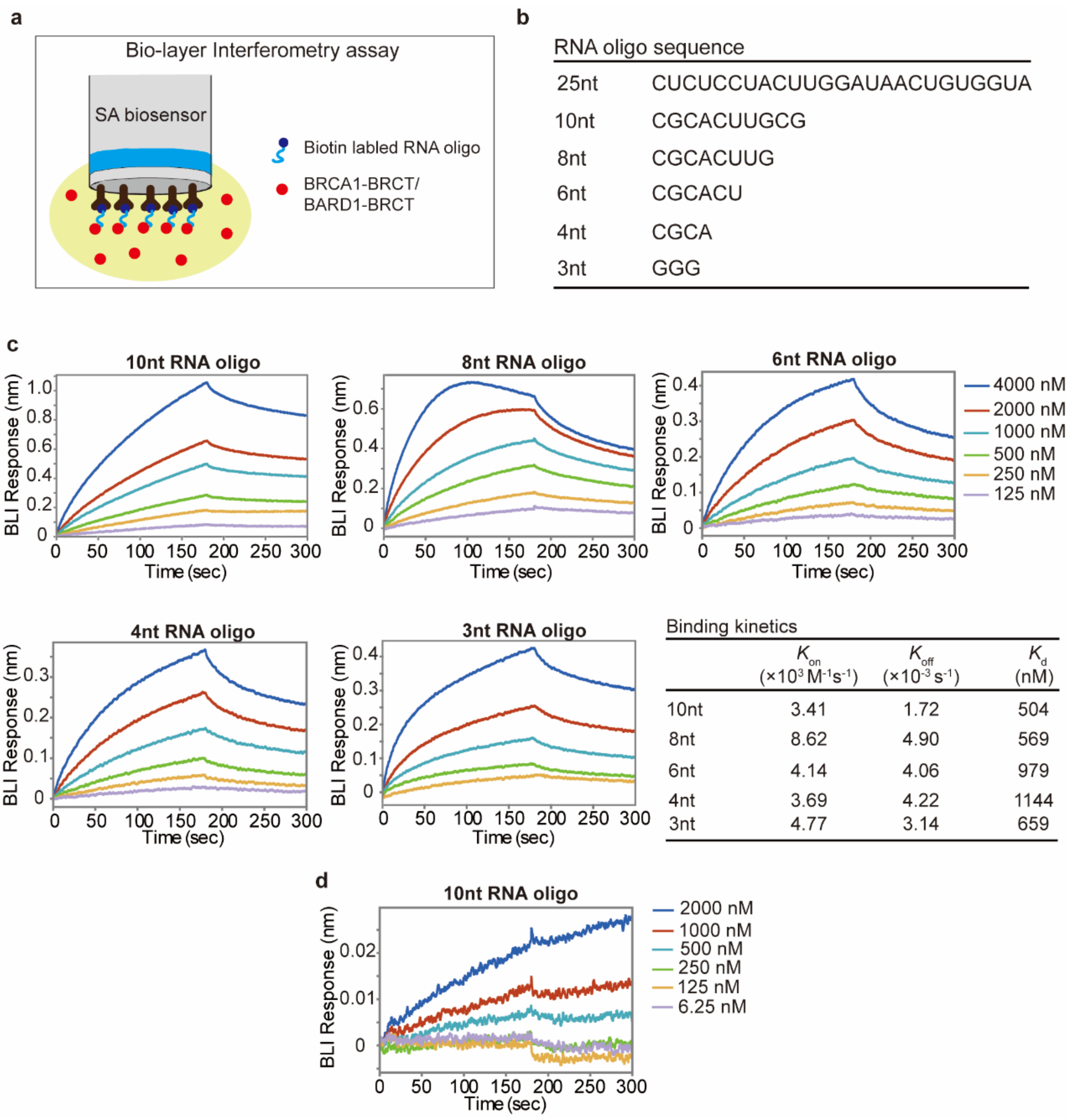

Supplementary Fig. S9 The BARD1 BRCT domain has a high affinity for RNA oligo.

(a) A schematic of bio-layer interferometry (BLI) using a SA biosensor to capture biotin-labeled RNA oligo, then interact with corresponding proteins. **(b)** The sequence of RNA oligo used to bind the BRCA1 BRCT or BARD1 BRCT. **(c)** BLI comparison of binding properties of the BARD1 BRCT to different lengths of RNA oligos. To measure binding properties, purified BARD1 BRCT was loaded onto the sensor at different concentrations. Fit two-phase (association then dissociation) nonlinear

regression curves based on the average of 3 experiments. **(d)** BLI assays were performed to examine the interaction between the BRCA1 BRCT and 10 nt RNA oligo.

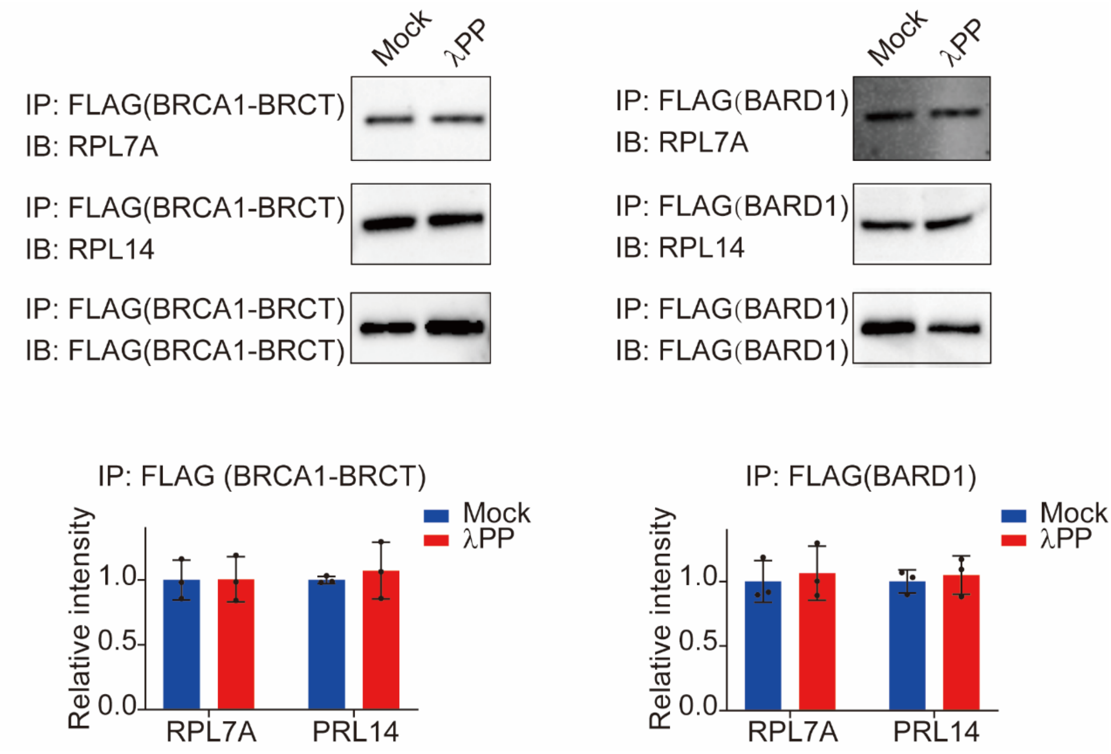

Supplementary Fig. S10 The interactions between the BRCA1/BARD1 complex and ribosomal proteins are independent of protein phosphorylation.

The cell lysates were examined with IP and Western blot with indicated antibodies. IPed samples were treated with or without λ phosphatase. The relative amount of RPL7A or RPL14 was calculated and shown in the histogram (bottom panel). Values are mean ± SD of three independent assays.

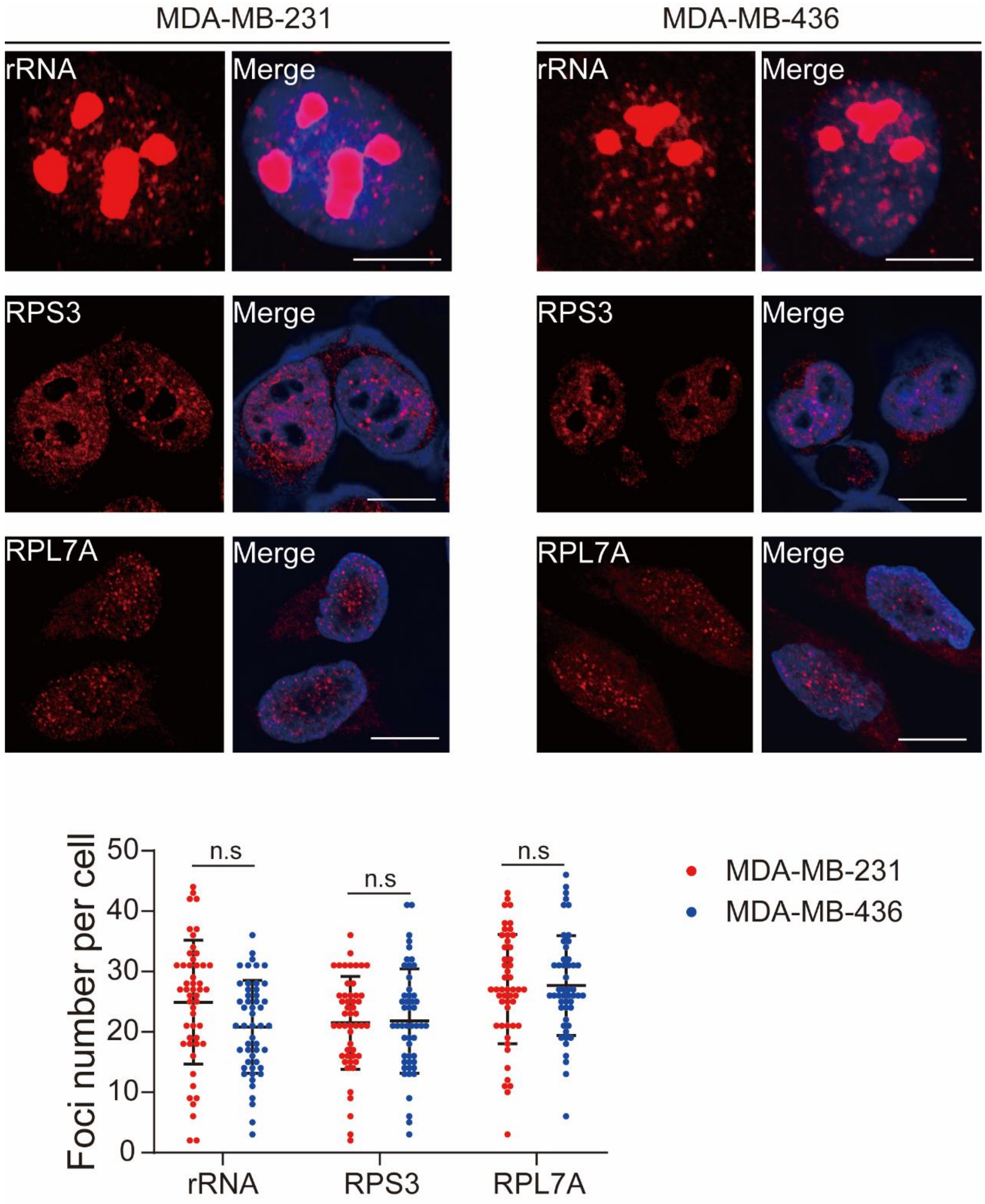

Supplementary Fig. S11 The pre-rRNP foci are not affected by the status of BRCA1.

The IRIF of pre-rRNP in both MDA-MB-231 and MDA-MB-436 cells were examined. Cells were treated with 10 Gy of IR. Pre-rRNA was examined by RNA probes. Ribosomal proteins were stained with indicated antibodies. Foci numbers were

analyzed on the bottom panel. n.s. $p>0.05$. The image bar is 10 μm.

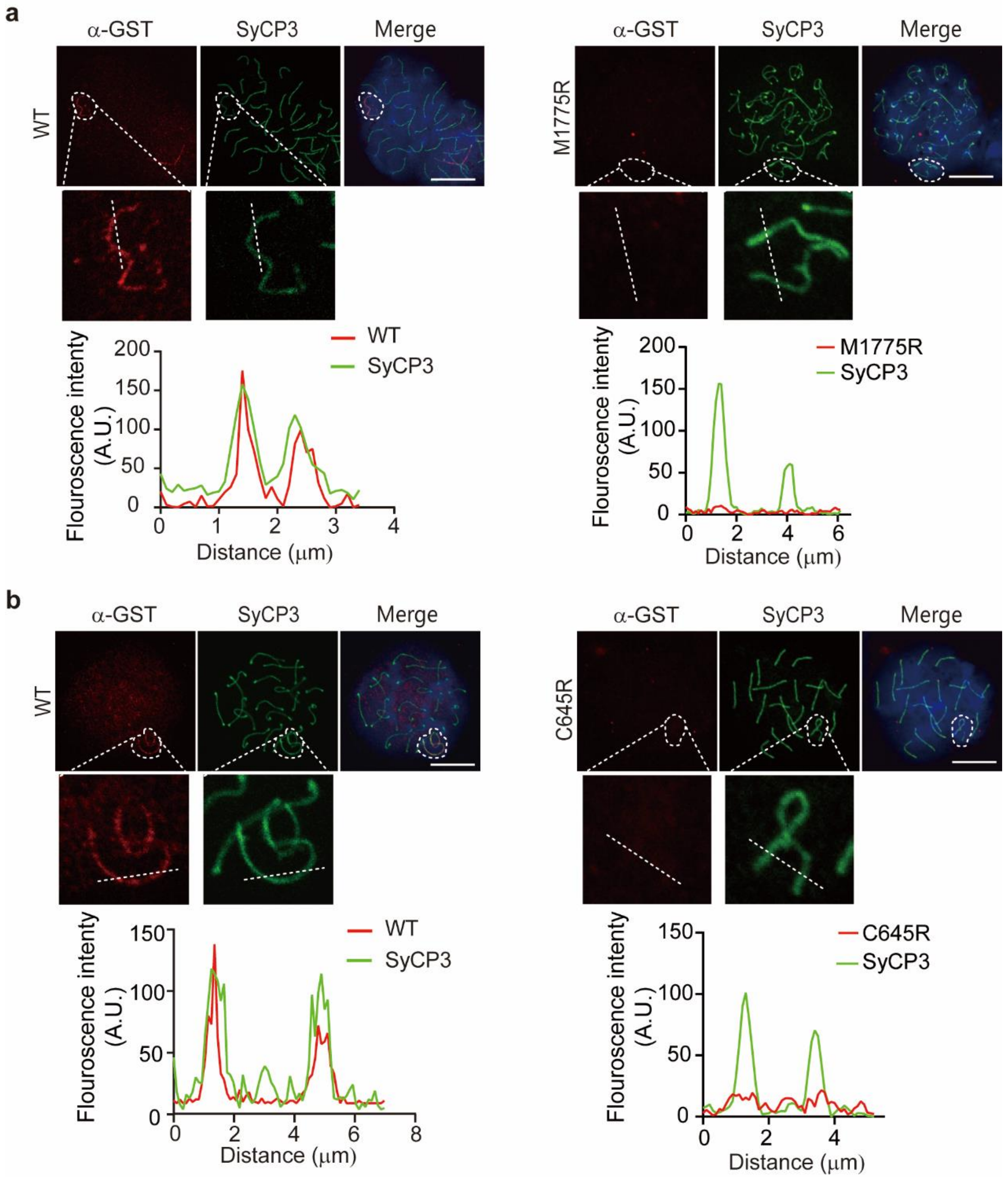

Supplementary Fig. S12 Mutant BRCA1 BRCT or BARD1 BRCT does not localize to the XY body.

(a) M1775R mutant of BRCA1-BRCT or **(b)** C645R mutant of BARD1-BRCT cannot be hybridized onto the unsynapsed region of the XY body. The image bar is 10 μm.

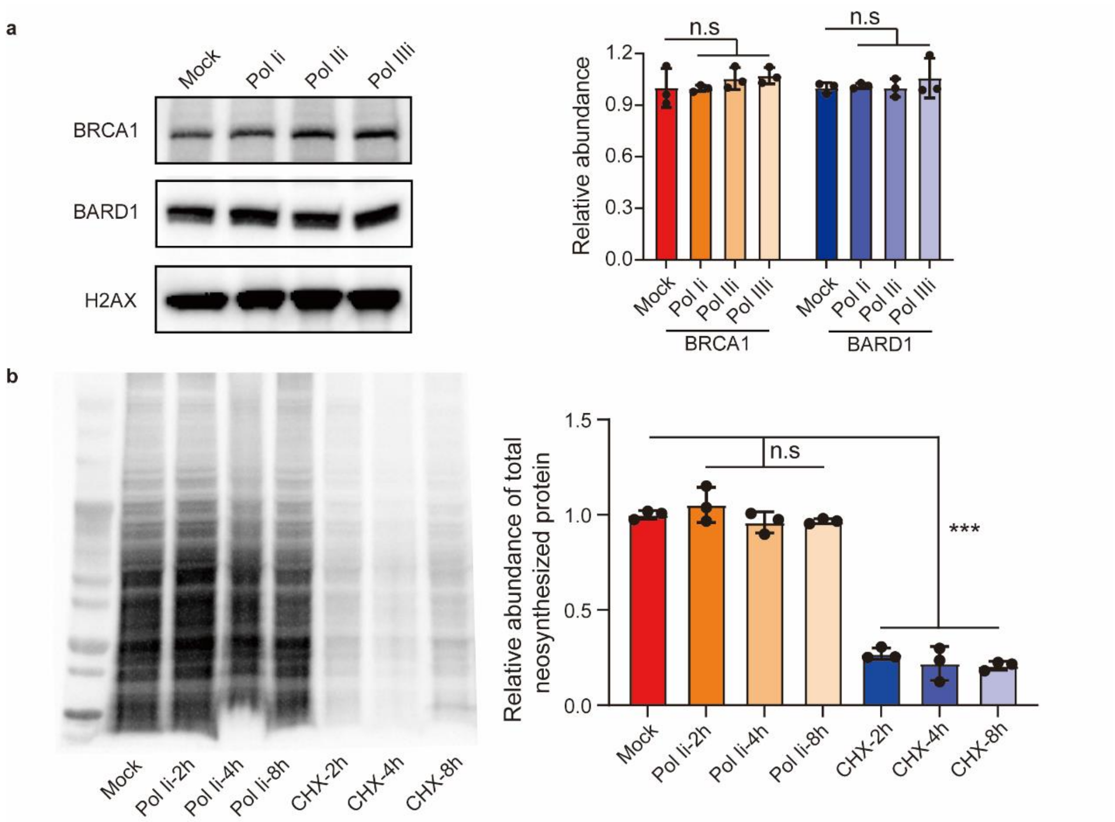

Supplementary Fig. S13 Transient pol Ii treatment has little impact on protein synthesis.

(a) Transient Pol Ii treatment has little effect on the protein expression of BRCA1, BARD1 and H2AX. Cells were treated with Pol I, Pol II and Pol III inhibitors for 2 h before lysis, DMSO treatment was used as mock. The cell lysates were examined with Western blot analysis. **(b)** Transient Pol Ii treatment has little effect on overall protein translation. Neosynthesized proteins were determined after mock (DMSO), pol Ii or cycloheximide (CHX) treatment. Values are mean ± SD of three independent assays. P-values were calculated using Student's t-test. n.s.: nonsignificant, ***$P < 0.001$.

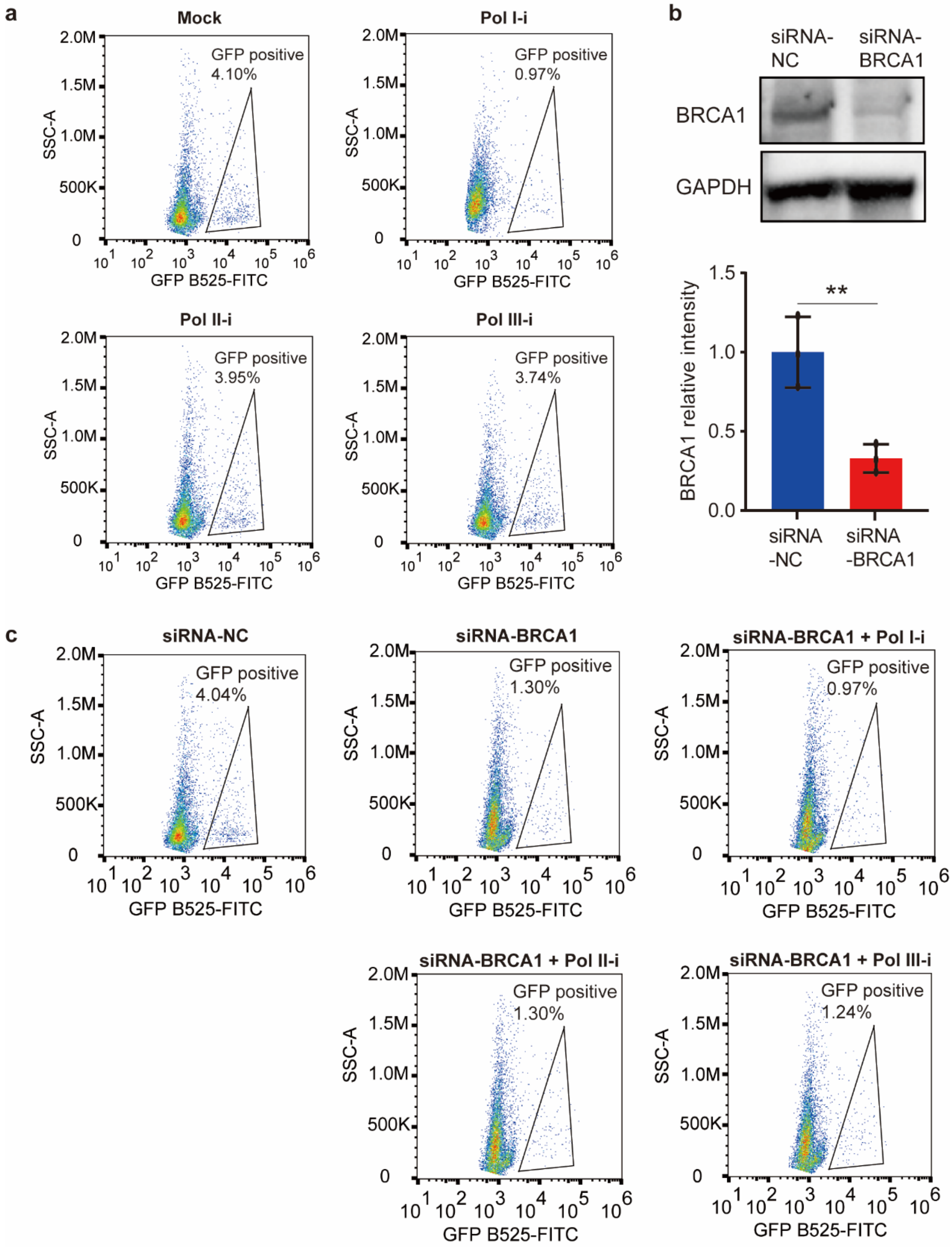

Supplementary Fig. S14 Pol Ii treatment impairs HR repair.

(a) Pol Ii treatment suppresses HR repair. DR-GFP reporter system was utilized to determine the HR efficiency. Following pol I, pol II or pol III inhibitor treatment (1 μM), GFP fluorescence in U2OS DR-GFP reporter cells were detected with flow

cytometry. **(b)** SiRNA targeting *BRCA1* drastically reduces the BRCA1 protein expression level. **(c)** Pol I is epistatic with BRCA1 on HR. Following knockdown BRCA1 by siRNA and pol I, pol II or pol III inhibitor treatment, HR repair efficiency were examined by DR-GFP reporter assays.

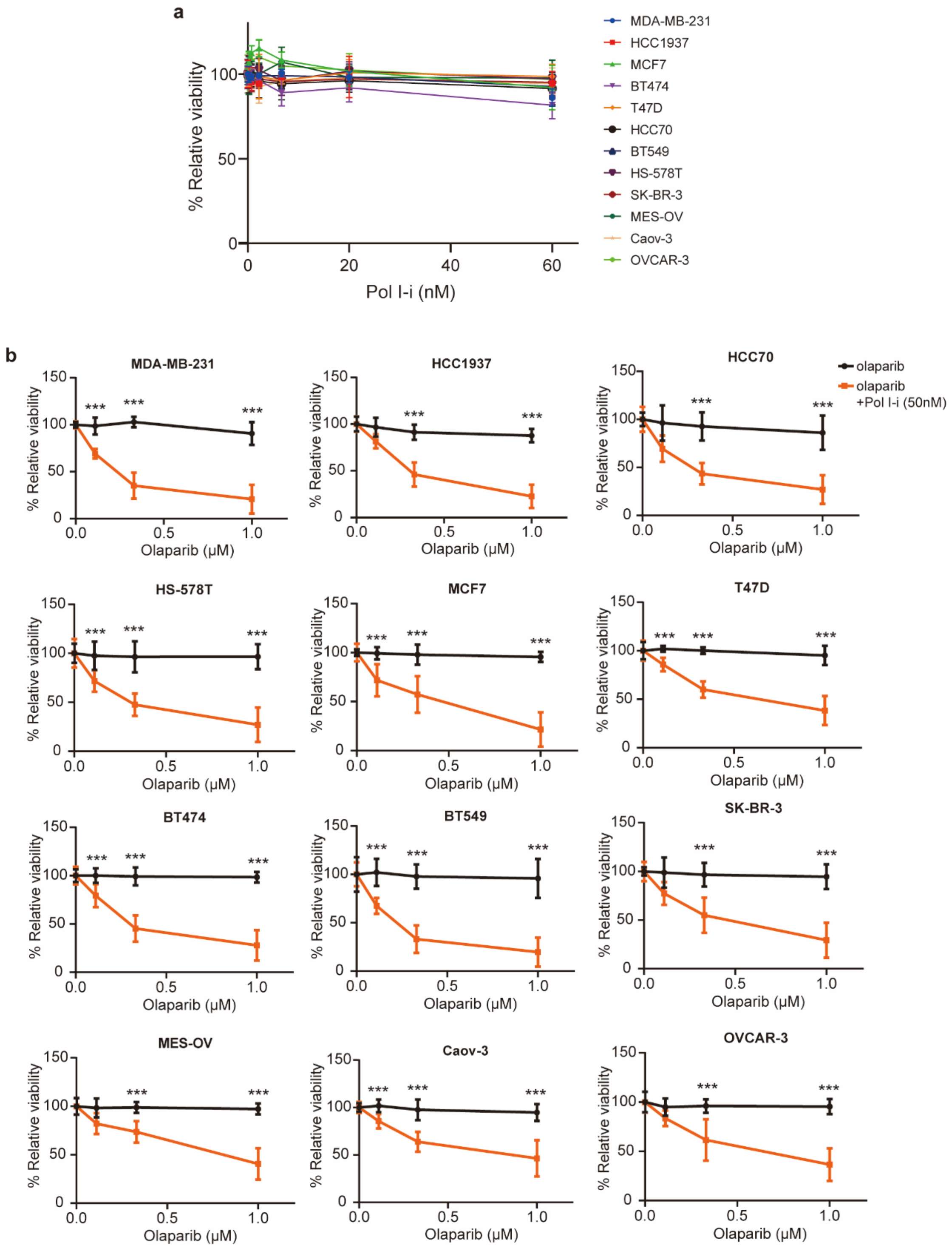

a
% Relative viability
Pol I-i (nM)
MDA-MB-231
HCC1937
MCF7
BT474
T47D
HCC70
BT549
HS-578T
SK-BR-3
MES-OV
Caov-3
OVCAR-3
b
MDA-MB-231
HCC1937
HCC70
HS-578T
MCF7
T47D
BT474
BT549
SK-BR-3
MES-OV
Caov-3
OVCAR-3
olaparib
olaparib +Pol I-i (50nM)
% Relative viability
Olaparib (μM)
***

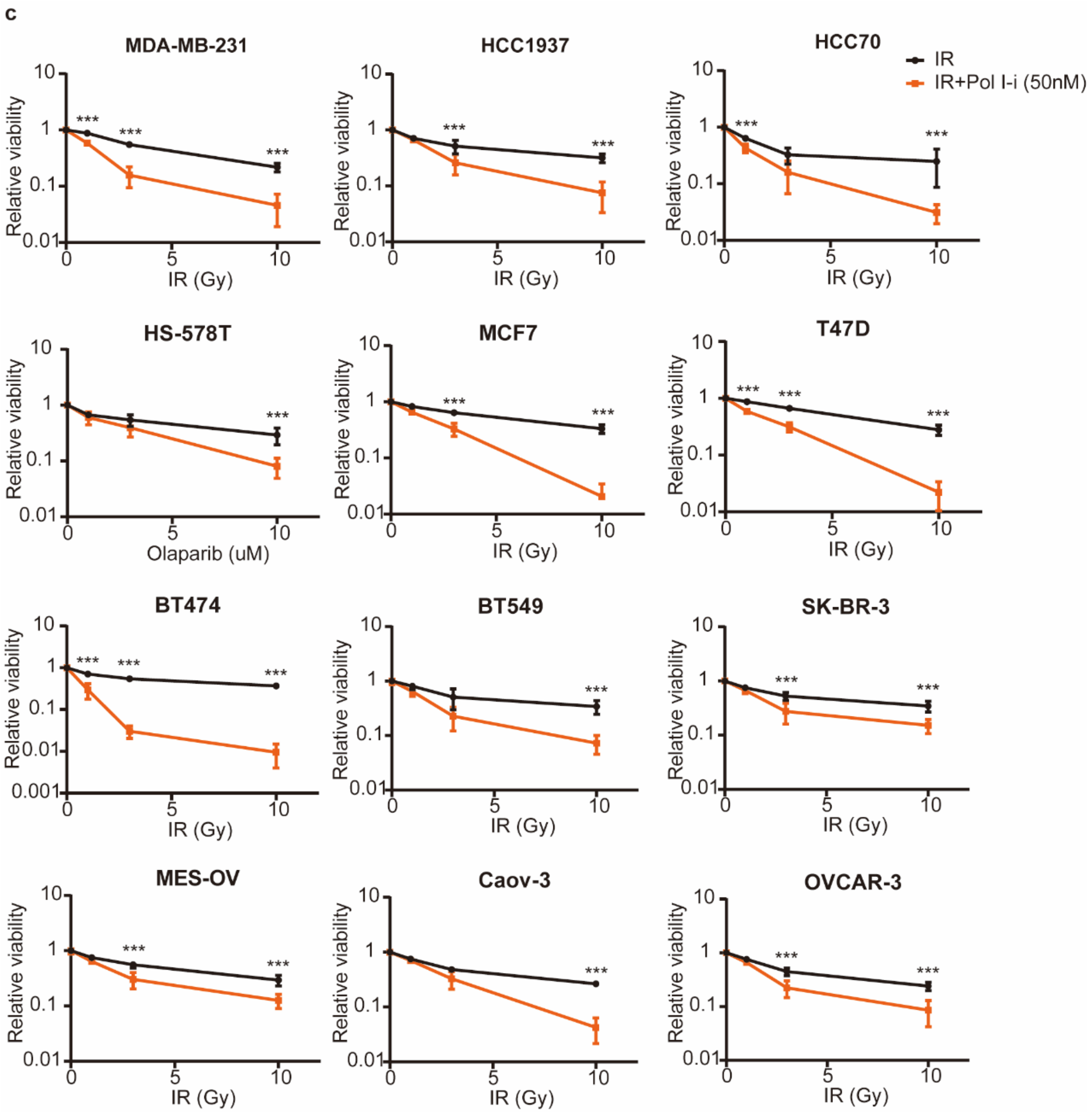

Supplementary Fig. S15 Pol I inhibitor treatment sensitizes tumor cells to PARP inhibitor or IR treatment.

(a) Low dose Pol Ii treatment does not suppress cancer cell growth. A panel of breast and ovarian cancer cells were treated with indicated dose of Pol Ii for 7 days. Cell viability was examined by CTG assays. **(b, c)** Pol Ii treatment (50nM) sensitizes breast and ovarian cancer cells to PARPi **(b)** or IR **(c)**. Values are mean ± SD of three different assays. P-values were calculated using Student's t-test. n.s.: nonsignificant, *P < 0.05, and ***P < 0.001.

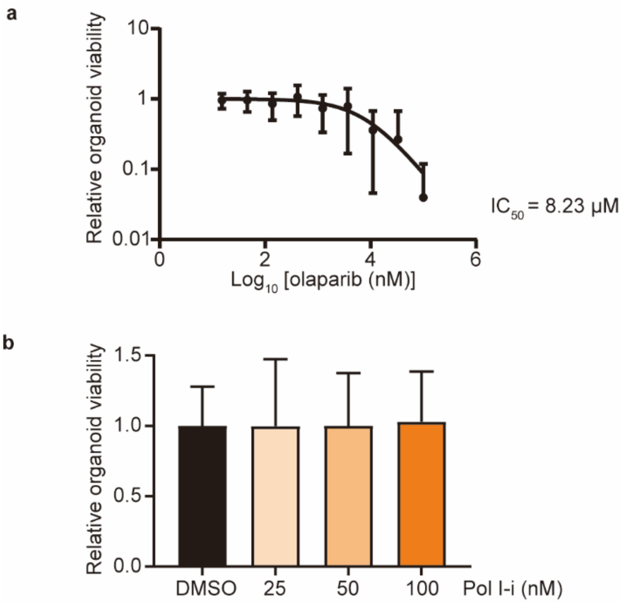

Supplementary Fig. S16 Monotherapy of Pol Ii or PARPi on TNBC organoid.

(a) IC_{50} of olaparib on TNBC organoid. **(b)** Low-dose Pol Ii treatment does not inhibit organoids growth. Organoids were treated with the indicated dose of Pol Ii for 7 days. Values are mean ± SD of three independent assays.

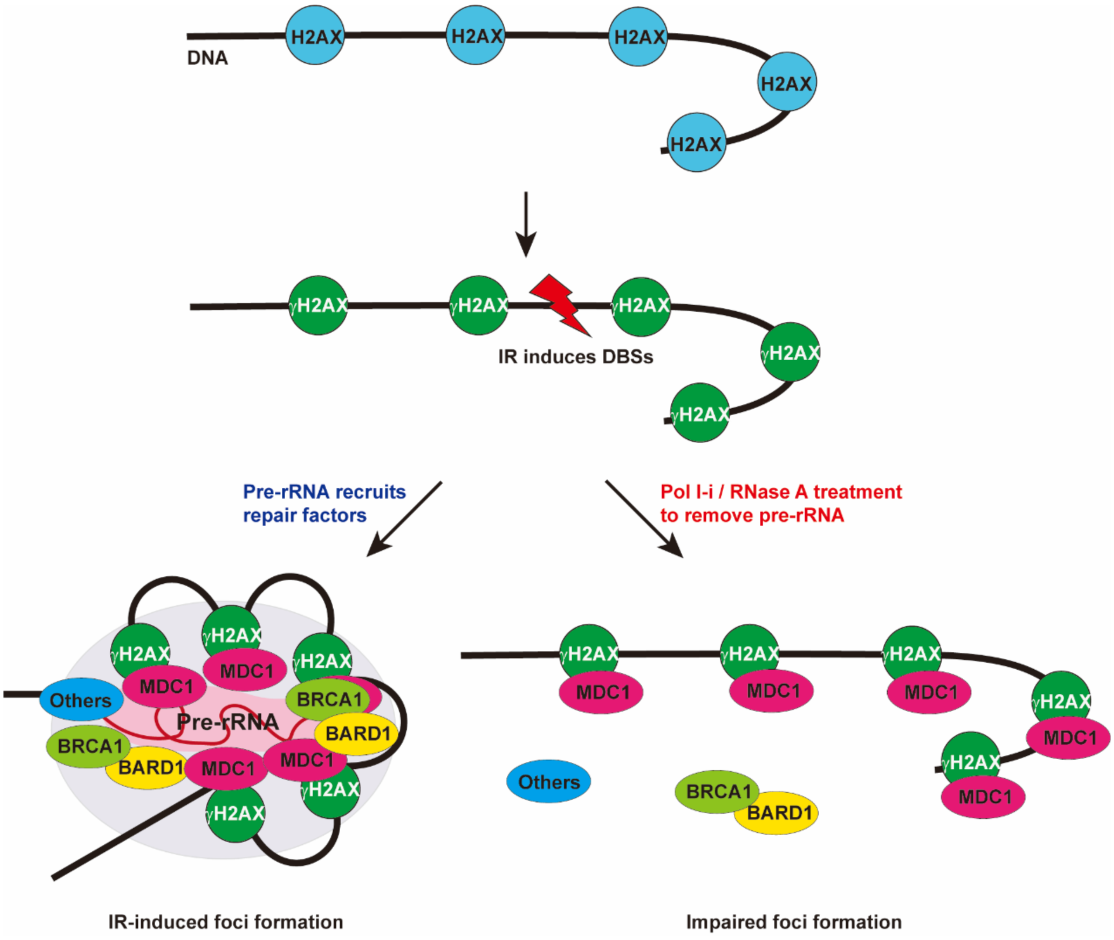

Supplementary Fig. S17 A working model of the functional interaction between γH2AX-MDC1 and pre-rRNA to mediate foci of the BRCA1-BARD1 complex.

Supplementary Table

Supplementary Table S3. Primers and probes sequence used in this manuscript.

Primer Name	Sequence
45S-qPCR-s	CCCACCCTCGGTGAGAAAAG
45S-qPCR-as	GGAAGCGGAGGAGGGTCCTC
18S-qPCR-s	GGCCCGAAGCGTTTACTTTG
18S-qPCR-as	GCGGCGCAATACGAATGCC
ITS1-qPCR-a	CGAGAGCCGGAGAACTCGG
ITS1-qPCR-as	GCCGACACCCACGTCGTC
ITS2-qPCR-s	CGGGCCCTGCGTGGTCAC
ITS2-qPCR-as	GGAGGAACCCGGACCGCAG
28S-qPCR-s	CACGAGACCGATAGTCAACAAG
28S-qPCR-as	AACGGGGGGCGGGAAAGATC
5.8S-qPCR-a	GACTCTTAGCGGTGGATCAC
5.8S-qPCR-as	AAGCGACGCTCAGACAGGC
BRCA1-s	CAAGGTCCAAAGCGAGCAAGAG
BRCA1-as	CACCACAGAAGCACCACACAGC
GAPDH-s	GTCTCCTCTGACTTCAACAGCG
GAPDH-as	ACCACCCTGTTGCTGTAGCCAA

Probe name	Sequence
ITS1-Cy3	CCTCGCCCTCCGGGCTCCGTTAATGATC
ITS2-Cy3	CTGCGAGGGAACCCCCAGCCGCGCA
5.8S-Cy3	CATCGACGCACGAGCCGAGTGATCCAC
28S-Cy3	TTAGATGGAGTTTACCACCC
18S-Cy3	CTGGCAGGATCAACCAGGTA
